# Pandemic-type failures in multivariate Brownian risk models

**DOI:** 10.1007/s10687-021-00424-4

**Published:** 2021-09-22

**Authors:** Krzysztof Dȩbicki, Enkelejd Hashorva, Nikolai Kriukov

**Affiliations:** 1 Mathematical Institute, University of Wrocław, pl. Grunwaldzki 2/4, 50-384 Wrocław, Poland; 2 Department of Actuarial Science, University of Lausanne, UNIL-Dorigny, 1015 Lausanne, Switzerland

**Keywords:** Multivariate Brownian risk model, Probability of multiple simultaneous failures, Simultaneous ruin probability, Failure time, Exact asymptotics, Pandemic-type events, Primary–60G15, Secondary–60G70

## Abstract

Modelling of multiple simultaneous failures in insurance, finance and other areas of applied probability is important especially from the point of view of pandemic-type events. A benchmark limiting model for the analysis of multiple failures is the classical *d*-dimensional Brownian risk model (Brm), see Delsing et al. (Methodol. Comput. Appl. Probab. **22**(3), 927–948 2020). From both theoretical and practical point of view, of interest is the calculation of the probability of multiple simultaneous failures in a given time horizon. The main findings of this contribution concern the approximation of the probability that at least *k* out of *d* components of Brm fail simultaneously. We derive both sharp bounds and asymptotic approximations of the probability of interest for the finite and the infinite time horizon. Our results extend previous findings of Dȩbicki et al. (J. Appl. Probab. **57**(2), 597–612 2020) and Dȩbicki et al. (Stoch. Proc. Appl. **128**(12), 4171–4206 2018).

## Introduction

In this paper we are interested in the probabilistic aspects of multiple simultaneous failures typically occurring due to pandemic-type events. A key benchmark risk model considered here is the *d*-dimensional Brownian risk model (Brm)

$$ \boldsymbol{R}(t, \boldsymbol{u})=(R_{1}(t,u_{1}),\ldots,R_{d}(t,u_{d}))^{\top}= \boldsymbol{u} {+ \boldsymbol{c} t- \boldsymbol{W}(t)}, \quad t\ge 0,$$

where ***c*** = (*c*_1_, … , *c*_*d*_)^⊤^, ***u*** = (*u*_1_, … , *u*_*d*_)^⊤^ are vectors in $\mathbb {R}^{d}$ and

$$\boldsymbol{W}(t)= {\Gamma} \boldsymbol B(t), \quad t\in \mathbb{R} ,$$

with Γ a *d* × *d* real-valued non-singular matrix and $\boldsymbol B(t)=(B_{1}(t) ,\ldots , B_{d}(t))^{\top } , t \in \mathbb {R}$ a *d*-dimensional Brownian motion with independent components which are standard Brownian motions.

By bold symbols we denote column vectors, operations with vectors are meant component-wise and *a****x*** = (*ax*_1_, … , *ax*_*d*_)^⊤^ for any scalar $a\in \mathbb {R}$ and any $\boldsymbol {x}\in \mathbb {R}^{d}$.

Indeed, Brm is a natural limiting model in many statistical applications. Moreover, as shown in Delsing et al. (2020) such a risk model appears naturally in insurance applications.Since Brm is a natural limiting model, it can be used as a benchmark for various complex models. Given the fundamental role of Brownian motion in applied probability and statistics, it is also of theoretical interest to study failure events arising from this model. Specifically, in this contribution we are interested in the behaviour of the probability of multiple simultaneous failures occurring in a given time horizon $[S,T] \subset [0, \infty ]$.

In our settings failures can be defined in various ways. Let us consider first the failure of a given component of our risk model. Namely, we say that the *i* th component of our Brm has a failure (or ruin occurs) if *R*_*i*_(*t*, *u*_*i*_)= *u*_*i*_+*c*_*i*_*t* − *W*_*i*_(*t*) < 0 for some *t* ∈ [*S*, *T*]. The extreme case of a catastrophic event is when *d* multiple simultaneous failures occurs. Typically, for pandemic-type events there are at least *k* components of the model with simultaneous failures and *k* is large with the extreme case *k* = *d*. In mathematical notation, for given positive integer *k* ≤ *d* of interest is the calculation of the following probability

$$ \begin{array}{@{}rcl@{}} \psi_k(S,T,\boldsymbol{u})&=& \mathbb{P} \left\{ \exists t\in[S,T], \exists \mathcal{I}\subset\{1,\ldots, d\},|\mathcal{I}|=k: \cap_{i\in \mathcal{I}} \{ R_i(t, { u_i}) < 0\} \right \} \\ &=& \mathbb{P} \left\{ \exists t\in[S,T], \exists \mathcal{I}\subset\{1,\ldots, d\},|\mathcal{I}| = k: {\cap_{i\in \mathcal{I}}} {\{}W_i(t)- c_i t\!>\!u_i{\}} \right \} , \end{array} $$

where $|\mathcal {I}|$ denotes the cardinality of the set $\mathcal {I}$. If *T* is finite, by the self-similarity property of the Brownian motion *ψ*_*k*_(*S*, *T*, ***u***) can be derived from the case *T* = 1, whereas $T=\infty $ has to be treated separately.

There are no results in the literature investigating *ψ*_*k*_(*S*, *T*, ***u***) for general *k*. The particular case *k* = *d*, for which *ψ*_*d*_(*S*, *T*, ***u***) coincides with the simultaneous ruin probability has been studies in different contexts, see e.g., Lieshout and Mandjes (2007), Avram et al. (2008a), Avram et al. (2008b), Dȩbicki et al. (2018), Ji and Robert (2018), Foss et al. (2017), Pan and Borovkov (2019), Borovkov and Palmowski (2019), Ji (2020), Hu and Jiang (2013), Samorodnitsky and Sun (2016), and Dombry and Rabehasaina (2017). The case *d* = 2 of Brm has been recently investigated in Dȩbicki et al. (2020).

Although the probability of multiple simultaneous failures seems very difficult to compute, our first result below, motivated by Korshunov and Wang (2020)[Thm 1.1], shows that *ψ*_*k*_(*S*, *T*, ***u***) can be bounded by the multivariate Gaussian survival probability, namely by

$$ p_{T}(\boldsymbol{u})= \mathbb{P} \left\{ {(W_{1}(T)- c_{1} T ,\ldots, W_{d}(T)- c_{d} T)} \in \boldsymbol E(\boldsymbol{u}) \right \} ,$$

where

1 $$ \boldsymbol E(\boldsymbol{u})=\underset{\underset{|\mathcal{I}|=k}{\mathcal{I}\subset\{1,\ldots, d\}}}{\bigcup}\boldsymbol{E_{\mathcal{I}}(\boldsymbol{u})}=\underset{\underset{|\mathcal{I}|=k}{\mathcal{I}\subset\{1,\ldots, d\}}}{\bigcup}{\{\boldsymbol{x}\in\mathbb{R}^d:\forall i\in \mathcal{I}:~\boldsymbol{x}_i\geq\boldsymbol{u}_i\}}. $$

When $u\to \infty $ we can approximate *p*_*T*_(***u***) utilising Laplace asymptotic method, see e.g., Korshunov et al. (2015), whereas for small and moderate values of *u* it can be calculated or simulated with sufficient accuracy. Our next result gives bounds for *ψ*_*k*_(*S*, *T*, ***u***) in terms of *p*_*T*_(***u***).

### **Theorem 1.1**

If the matrix Γ is non-singular, then for any positive integer *k* ≤ *d*, all constants $ 0\leq S < T< \infty $ and all $\boldsymbol {c},\boldsymbol {u}\in \mathbb {R}^{d}$

2 $$ p_T(\boldsymbol{u}) \le \psi_k({S},T, \boldsymbol{u}) \le K p_T(\boldsymbol{u}), $$

where $K= 1/\min \limits _{\substack {\mathcal {I}\subset \{1,\ldots , d\},|\mathcal {I}|=k}}\mathbb {P} \left \{ \forall _{i\in \mathcal {I}}: W_{i}(T)> \max \limits (0,c_{i} T) \right \} >0$.

The bounds in Eq. 2 indicate that it might be possible to derive an approximations of *ψ*_*k*_(*S*, *T*, ***u***) for large threshold ***u***, which has been already shown for *k* = *d* = 2 in Dȩbicki et al. (2020). In this paper we consider the general case *k* ≤ *d*, *d* > 2 discussing both the finite time interval (i.e., *T* = 1) and the infinite time horizon case with $T=\infty $ extending the results of Dȩbicki et al. (2018) where *d* = *k* is considered.

In Section 2 we explain the main ideas that lead to the approximation of *ψ*_*k*_(*S*, *T*, ***u***). Section 3 discusses some interesting special cases, whereas the proofs are postponed to Section 4. Some technical calculations are displayed in Appendix.

## Main results

In this section ***W***(*t*), *t* ≥ 0 is as in the Introduction and for a given positive integer *k* ≤ *d* we shall investigate the approximation of *ψ*_*k*_(*S*, *T*, ***u***) where we fix ***u*** = ***a****u*, with ***a*** in $\mathbb {R}^{d}\setminus (-\infty ,0]^{d}$ and *u* is sufficiently large.

Let hereafter $\mathcal {I}$ denote a non-empty index set of {1, … , *d*}. For a given vector, say $\boldsymbol {x}\in \mathbb {R}^{d}$ we shall write $\boldsymbol {x}_{\mathcal {I}}$ to denote a subvector of ***x*** obtained by dropping its components not in $\mathcal {I}$. Set next

$$ \psi_{\mathcal{I}}(S,T, \boldsymbol {a}_{\mathcal{I}} u)= \mathbb{P} \left\{ \exists {t\in [S,T]}: A_{\mathcal{I}}(t) \right \} ,$$

with

3 $$ A_{\mathcal{I}} (t)=\{ \boldsymbol{W}(t)- \boldsymbol{c} t\in\boldsymbol E_{\mathcal{I}}(\boldsymbol {a} u) \}=\{ \forall i\in\mathcal{I}:\ ~W_i(t)- c_it\geq a_i u \}, $$

where $\boldsymbol {E}_{\mathcal {I}}(\boldsymbol {a} u)$ was defined in Eq. 1.

In vector notation for any $u\in \mathbb {R}$

$$\psi_k(S,T,\boldsymbol {a} u) = \mathbb{P} \left\{ \exists t\in[S,T]:~\underset{\underset{|\mathcal{I}|=k}{\mathcal{I}\subset\{1,\ldots, d\}}}{\bigcup}A_{\mathcal{I}}(t) \right \} = \mathbb{P} \left\{ \underset{\underset{{|\mathcal{I}|=k}}{\mathcal{I}\subset\{1,\ldots, d\}}}{\bigcup}\left\{\exists t\in[S,T]:~A_{\mathcal{I}}(t)\right\} \right \}. $$

The following lower bound (by Bonferroni inequality)

4 $$ \psi_k(S, T,\boldsymbol {a} u) \ge \underset{\underset{|\mathcal{I}|=k}{\mathcal{I}\subset\{1,\ldots, d\}}}{\sum} \psi_{\mathcal{I}} (S,T,\boldsymbol {a}_{\mathcal{I}} u) -\underset{\begin{array}{cc}\mathcal{I},\mathcal{J}\subset\{1,\ldots, d\}\\|\mathcal{I}|=|\mathcal{J}|=k\\\mathcal{I}\not=\mathcal{J} \end{array}}{\sum}\mathbb{P} \left\{ \exists t,s\in[S,T]:~A_{\mathcal{I}}(t)\cap A_{\mathcal{J}}(s) \right \} $$

together with the upper bound

5 $$ \begin{array}{@{}rcl@{}} \psi_k(S, T,\boldsymbol {a} u) \le \underset{\underset{{|\mathcal{I}|=k}}{\mathcal{I}\subset\{1,\ldots, d\}}}{\sum} \psi_{\mathcal{I}} (S,T,\boldsymbol {a}_{\mathcal{I}} u) \end{array} $$

are crucial for the derivation of the exact asymptotics of *ψ*_*k*_(*S*, *T*, ***a****u*) as $u\to \infty $. As we shall show below, the upper bound (5) turns out to be exact asymptotically as $u\to \infty $. The following theorem constitutes the main finding of this contribution.

### **Theorem 2.1**

Suppose that the square *d* × *d* real-valued matrix Γ is non-singular. If ***a*** has no more than *k* − 1 non-positive components, where *k* ≤ *d* is a positive integer, then for all $ 0 \leq S< T < \infty , \boldsymbol {c}\in \mathbb {R}^{d} $

6 $$ \psi_k(S, T, \boldsymbol {a} u) \sim {\sum}_{\substack{\mathcal{I}\subset\{1,\ldots, d\}\\|\mathcal{I}|=k}} \psi_{\mathcal{I}} (0,T,\boldsymbol {a}_{\mathcal{I}} u) , \quad u\to \infty. $$

Moreover, Eq. 6 holds also if $T=\infty $, provided that ***c*** and ***a*** + ***c****t* have no more than *k* − 1 non-positive components for all *t* ≥ 0.

Essentially, the above result is the claim that the second term in the Bonferroni lower bound (4) is asymptotically negligible. In order to prove that, the asymptotics of $ \psi _{\left \lvert \mathcal {I} \right \rvert } (S,T,\boldsymbol {a}_{\mathcal {I}} u)$ has to be derived. For the special case that $\mathcal {I}$ has only two elements and *S* = 0, its approximation has been obtained in Dȩbicki et al. (2020). Note in passing that the assumption in Theorem 2.1 that ***a*** has no more than *k* − 1 non-positive components excludes the case that there exists a set $\mathcal {I} \subset \{1,\ldots , d\}, \ |\mathcal {I}|=k$ such that $\psi _{\mathcal {I}} (0,T,\boldsymbol {a}_{\mathcal {I}} u)$ does not tend to 0 as $u\to \infty $, which due to its non-rare event nature is out of interest in this contribution.

The next result extends the findings of Dȩbicki et al. (2020) to the case *d* > 2. For notational simplicity we consider the case $\mathcal {I}$ has *d* elements and thus avoid indexing by $\mathcal {I}$. Recall that in our model ***W***(*t*) = Γ***B***(*t*) where ***B***(*t*) has independent standard Brownian motion components and Γ is a *d* × *d* non-singular real-valued matrix. Consequently Σ = ΓΓ^⊤^ is a positive definite matrix. Hereafter $\boldsymbol 0 \in \mathbb {R}^{d}$ is the column vector with all elements equal 0. Denote by π_Σ_(***a***) the quadratic programming problem:

$$ \text{minimise } \boldsymbol{x}^{\top}{\Sigma}^{-1} \boldsymbol{x}, \text{ for all } \boldsymbol{x}\ge \boldsymbol {a}. $$

Its unique solution $\tilde { \boldsymbol {a}}$ is such that

7 $$ \tilde{\boldsymbol {a}}_{I}= \boldsymbol {a}_{I}, \ \ ({\Sigma}_{II})^{-1} \boldsymbol{a}_{I}>\boldsymbol{0}_{I}, \ \ \tilde{\boldsymbol {a}}_{J}= {\Sigma}_{JI} ({\Sigma}_{II} )^{-1} \boldsymbol {a}_{I} \ge \boldsymbol {a}_{J}, $$

where $\tilde {\boldsymbol {a}}_{J}$ is defined if *J* = {1, … , *d*}∖ *I* is non-empty. The index set *I* is unique with $m=\left \lvert I \right \rvert \ge 1$ elements, see the next lemma (or Dȩbicki et al. (2018)[Lem 2.1]) for more details.

### **Lemma 2.2**

Let Σ be a *d* × *d* positive definite matrix and let $\boldsymbol {a} \in \mathbb {R}^{d} \setminus (-\infty , 0]^{d} $. π_Σ_(***a***) has a unique solution $\tilde {\textbf {a}}$ given in (7) with *I* a unique non-empty index set with *m* ≤ *d* elements such that

8 $$ \min_{\boldsymbol{x} \ge \boldsymbol{a}}\boldsymbol{x}^{\top} {\Sigma}^{-1} \boldsymbol{x}= \tilde{\textbf{a}}^{\top} {\Sigma}^{-1} \tilde{\textbf{a}} = \boldsymbol{a}_{I}^{\top} ({\Sigma}_{II})^{-1}\boldsymbol{a}_{I}>0, $$

9 $$ \boldsymbol{x}^{\top} {\Sigma}^{-1} \tilde{\textbf{a}}= \boldsymbol{x}_F^{\top} ({\Sigma}_{FF})^{-1} {\tilde{\textbf{a}}_F}, \quad \forall \boldsymbol{x}\in \mathbb{R}^d $$

for any index set *F* ⊂{1, … , *d*} containing *I*. Further if $\boldsymbol {a}= {(a ,\ldots , a)^{\top } , a}\in (0,\infty )$, then $ 2 \le \left \lvert I \right \rvert \le d$.

In the following we set

$$ \boldsymbol \lambda = {\Sigma}^{-1} \tilde{\boldsymbol {a}}. $$

In view of the above lemma

10 $$ \boldsymbol \lambda_I= ({\Sigma}_{II})^{-1} \boldsymbol {a}_I> \boldsymbol 0_I, \ \ \boldsymbol \lambda_J \ge \boldsymbol 0_J, $$

with the convention that when *J* is empty the indexing should be disregarded so that the last inequality above is irrelevant.

The next theorem extends the main result in Dȩbicki et al. (2020) and further complements findings presented in Theorem 2.1 showing that the simultaneous ruin probability (i.e., *k* = *d*) behaves up to some constant, asymptotically as $u\to \infty $ the same as *p*_*T*_(***u***). For notational simplicity and without loss of generality we consider next *T* = 1.

### **Theorem 2.3**

If $\boldsymbol {a} \in \mathbb {R}^{d}$ has at least one positive component and Γ is non-singular, then for all *S* ∈ [0,1)

11 $$ \psi_d (S,1, \boldsymbol {a} u) \sim C(\boldsymbol {a}) p_1(\boldsymbol {a} u) , \quad u\to \infty, $$

where $C(\boldsymbol {a})= {\prod }_{i\in I} \lambda _{i} {\int \limits }_{\mathbb {R}^{m}} \mathbb {P} \left \{ \exists _{t\ge 0}:\boldsymbol {W}_{I}(t)-t\boldsymbol {a}_{I}>\boldsymbol {x}_{I} \right \} e^{\boldsymbol \lambda ^{\top }_{I} \boldsymbol {x}_{I}} \mathrm {d}\boldsymbol {x}_{I} \in (0,\infty )$.

### *Remark 2.4*

i) By Lemma 4.6 below taking *T* = 1 therein (hereafter *φ* denotes the probability density function (pdf) of Γ***B***(1))

12 $$ p_1(\boldsymbol {a} u) =\mathbb{P} \left\{ \boldsymbol{W}(1)-\boldsymbol{c} >u\boldsymbol {a} \right \} \sim {\prod}_{i\in I} \lambda_i^{-1}\mathbb{P} \left\{ \boldsymbol{W}_U(1)> \boldsymbol{c}_U \lvert \boldsymbol{W}_I(1)> \boldsymbol{c}_I \right \} u^{-\left\lvert I \right\rvert}\varphi(u\tilde{\boldsymbol {a}}+\boldsymbol{c}) $$

as $u\to \infty $, where $\boldsymbol \lambda = {\Sigma }^{-1} \tilde {\boldsymbol {a}}$ and if *J* = {1, … , *d*}∖ *I* is non-empty, then $U=\{j\in J:\tilde { a}_{j}= a_{j}\}$. When *J* is empty the conditional probability related to *U* above is set to 1.

ii) Combining Theorems 2.1 and 2.3 for all *S* ∈ [0,1) and all $\boldsymbol {a} \in \mathbb {R}^{d}$ with no more than *k* − 1 non-positive components we have

13 $$ \psi_k(S, 1,\boldsymbol {a} u) \sim \underset{\underset{|\mathcal{I}|=k}{\mathcal{I}\subset\{1,\ldots, d\}}}{\sum} C(\boldsymbol {a}_{\mathcal{I}}) \psi_{\left\lvert \mathcal{I} \right\rvert} (0,1,\boldsymbol {a}_{\mathcal{I}} u) \sim C \mathbb{P} \left\{ \forall_{i \in \mathcal{I}^{*}}: W_i(1) > ua_i+ c_i \right \} , \quad u\to \infty $$

for some *C* > 0 and some $\mathcal {I}^{*}\subset \{1 ,\ldots , d\}$ with *k* elements.

iii) Comparing the results of Theorem 2.3 and Dȩbicki et al. (2018) we obtain
  $$ \limsup_{u\to \infty} \frac{ (-\ln \psi_{k}(S_{1}, 1,\boldsymbol {a} u))^{1/2}}{ - \ln \psi_{k}(S_{2}, \infty,\boldsymbol {a} u) }< \infty $$
  for all $ S_{1}\in [0,T], S_{2}\in [0,\infty )$.

iv) Define the failure time (consider for simplicity *k* = *d*) for our multidimensional model by

$$\tau(u)=\inf\{t\ge 0:\boldsymbol{W}(t)-t\boldsymbol{c}>\boldsymbol {a} u\},\qquad u>0. $$

If ***a*** has at least one positive component, then for all *T* > *S* ≥ 0, *x* > 0

14 $$ \lim_{u \to \infty} \mathbb{P} \left\{ u^2(T-\tau(u))\geq x|\tau(u) {\in [S,T]} \right \} = {e^{-x\frac{\tilde{\boldsymbol {a}}^{\top}{\Sigma}^{-1}\tilde{\boldsymbol {a}}}{2T^2}}}, $$

see the proof in Section 4.

## Examples

In order to illustrate our findings we shall consider three examples assuming that ΓΓ^⊤^ is a positive definite correlation matrix. The first example is dedicated to the simplest case *k* = 1. In the second one we discuss *k* = 2 restricting ***a*** to have all components equal to 1 followed then by the last example where only the assumption ΓΓ^⊤^ is an equi-correlated correlation matrix is imposed. In this section *T* = 1 and *S* ∈ [0,1) is fixed.

### *Example 1* (*k* = 1)

: Suppose that ***a*** has all components positive. In view of Theorem 2.1 we have that

$$\psi_{k}(S,1, \boldsymbol {a} u) \sim {\sum}_{i=1}^{d} \psi_{\{i\}}(0,1, a_{i}u)$$

as $u\to \infty $. Note that for any positive integer *i* ≤ *d*

$$\psi_{\{i\}}(0, 1, a_{i} u) = \mathbb{P} \left\{ \exists_{t\in [0,1]}: B(t)- c_{i} t > a_{i} u \right \} , $$

where *B* is a standard Brownian motion. It follows easily that

$$\psi_{k}(S,1, \boldsymbol {a} u) \sim 2{\sum}_{i=1}^{d}\mathbb{P} \left\{ B(1)> a_{i} u+c_{i} \right \} , \quad u\to \infty.$$

### *Example 2* (*k* = 2 and ***a*** = ***1***)

: Suppose next *k* = 2 and ***a*** has all components equal 1. By Theorems 2.1 and 2.3 we have that

$$\psi_{k}(S,1,\boldsymbol 1 u)\sim{\sum}_{\{i,j\}\subset\{1,\ldots, d\}}C_{i,j}(\boldsymbol 1)\mathbb{P} \left\{ \min_{k\in\{i,j\}}(W_{k}(1)-c_{k})>u) \right \} $$

as $u\to \infty $, where $\boldsymbol 1 \in \mathbb {R}^{d}$ has all components equal to 1. Using further Remark 2.4 we obtain

$${\mathbb{P} \left\{\min_{k\in\{i,j\}}(W_k(1)-c_k)>u)\right \} } \sim \frac{u^{-2}}{(1-\rho_{i,j})^2\sqrt{2\pi(1-\rho_{i,j}^2)}}e^{-\frac{u^2}{1+\rho_{i,j}} -{\frac{(c_i+c_j)u}{1+\rho_{i,j}}} -\frac{c_i^2-2\rho_{i,j}c_ic_j+c_j^2}{2(1-\rho^2_{i,j})}}, \quad u\to \infty. $$

Here we set *ρ*_*i*, *j*_ = *corr*(*W*_*i*_(1), *W*_*j*_(1)). Consequently, if $\rho _{i,j}>\rho _{i^{*},j^{*}}$, then as $u\to \infty $

$$ \mathbb{P} \left\{ \min_{k\in\{i^{*},j^{*}\}}(W_{k}(1)-c_{k}>u) \right \} =o\left( \mathbb{P} \left\{ \min_{k\in\{i,j\}}(W_{k}(1)-c_{k}>u) \right \} \right).$$

The same holds also if $\rho _{i,j}=\rho _{i^{*},j^{*}}$ and $c_{i}+c_{j}>c_{i^{*}}+c_{j^{*}}$. If we denote by *τ* the maximum of all *ρ*_*i*, *j*_’s and by *c*_∗_ the maximum of *c*_*i*_ + *c*_*j*_ for all *i*, *j*’s such that *ρ*_*i*, *j*_ = *τ*, then we conclude that

$$\psi_{k}(S,1,\boldsymbol au)\sim {\sum}_{i,j \in \{1,\ldots, d\}, \rho_{i,j}=\tau,~c_{i}+c_{j}=c_{*}}C_{i,j}(\boldsymbol 1)\mathbb{P} \left\{ \min_{k\in\{i,j\}}(W_{k}(1)-c_{k}>u) \right \} . $$

Note that in this case *C*_*i*, *j*_(***1***) does not depend on *i* and *j* and is equals to

$$(1-\tau)^{2}{\int}_{\mathbb{R}^{2}}\mathbb{P} \left\{ \exists_{t\ge 0}:B_{1}(t)-t>x,B_{2}(t)-t>y \right \} e^{(1-\tau^{2})(x+y)}\mathrm{d} x\mathrm{d} y,$$

where (*B*_1_(*t*), *B*_2_(*t*)), *t* ≥ 0 is a 2-dimensional Gaussian process with *B*_*i*_’s being standard Brownian motions with constant correlation *τ*. Consequently, as $u\to \infty $

$$\psi_{2}(S,1,\boldsymbol 1u)\sim C_*u^{-2}e^{-\frac{u^2}{1+\tau} -\frac{c_*u}{2(1{+}\tau)}}, $$

where

$$ \begin{array}{@{}rcl@{}}C_*&=&\frac{e^{-\frac{c_*^{2}}{2(1-{\tau}^2)}}}{\sqrt{2\pi(1-{\tau}^2)}} {\sum}_{i,j \in \{1,\ldots, d\}, \rho_{i,j}=\tau,~c_i+c_j=c_*}e^{\frac{c_ic_j}{1-\tau}}\\ && \times {\int}_{\mathbb{R}^2}\mathbb{P} \left\{ \exists_{t\ge 0}:B_1(t)-t>x,B_2(t)-t>y \right \} e^{(1-{\tau}^2)(x+y)}\mathrm{d} x\mathrm{d} y \in (0,\infty). \end{array} $$

### *Example 3* (Equi-correlated risk model)

: We consider the matrix Γ such that Σ = ΓΓ^⊤^ is an equi-correlated non-singular correlation matrix with off-diagonal entries equal to *ρ* ∈ (− 1/(*d* − 1),1). Let $\boldsymbol {a}\in \mathbb {R}^{d}$ with at least one positive component and assume for simplicity that its components are ordered, i.e., *a*_1_ ≥ *a*_2_ ≥⋯ ≥ *a*_*d*_ and thus *a*_1_ > 0. The inverse of Σ equals

$$ \left[J_{d} - \boldsymbol 1 \boldsymbol 1^{\top} \frac{ \rho }{ 1+ \rho(d-1)} \right] \frac{1}{ 1-\rho},$$

where *J*_*d*_ is the identity matrix. First we determine the index set *I* corresponding to the unique solution of π_Σ_(***a***). We have for this case that *I* with *m* elements is unique and in view of Eq. 7

15 $$ \boldsymbol \lambda_I= ({\Sigma}_{II})^{-1} \boldsymbol {a}_I= \frac 1 {1- \rho}\left[ \boldsymbol {a}_I - \rho \frac{{\sum}_{i\in I} a_i}{1+ \rho(m-1)} \boldsymbol 1_I\right] > \boldsymbol 0_I, $$

with $\boldsymbol 0 \in \mathbb {R}^{d}$ the origin. From the above $m=\left \lvert I \right \rvert =d$ if and only if

$$a_{d}> \rho \frac{{\sum}_{i=1}^{d} a_{i}}{1+ \rho(d-1)},$$

which holds in the particular case that all *a*_*i*_’s are equal and positive.

When the above does not hold, the second condition on the index set *I* given in Eq. 7 reads

$$ {\Sigma}_{JI}{\Sigma}_{II}^{-1} \boldsymbol {a}_{I}= \rho (\boldsymbol 1 \boldsymbol 1^{\top} )_{JI} {\Sigma}_{II}^{-1} \boldsymbol {a}_{I} \ge \boldsymbol {a}_{J}.$$

Next, suppose that $a_{i}=a>0, c_{i}=c\in \mathbb {R}$ for all *i* ≤ *d*. In view of Eq. 13 for any positive integer *k* ≤ *d* and any *S* ∈ [0,1) we have

16 $$ \psi_k(S, 1,\boldsymbol {a} u) \sim C\mathbb{P} \left\{ \forall_{i \le k}: W_i(1) > ua+ c \right \} , \quad u\to \infty, $$

where (set *I* = {1, … , *k*})

$$ C= \frac{d!}{k! (d-k)!} {\prod}_{i\in I} \lambda_{i} {\int}_{\mathbb{R}^{k}} \mathbb{P} \left\{ \exists_{t\ge 0}:\boldsymbol{W}_{I}(t)-t\boldsymbol {a}_{I}>\boldsymbol{x}_{I} \right \} e^{\boldsymbol \lambda_{I}^{\top} x_{I}} \mathrm{d}\boldsymbol{x}_{I} \in (0,\infty). $$

Note that the case *ρ* = 0 is treated in Bai et al. (2018)[Prop. 3.6] and follows as a special case of this example.

## Proofs

### Proof of Theorem 1.1

Our proof below is based on the idea of the proof of Korshunov and Wang (2020)[Thm 1.1], where ***c*** has zero components, *k* = *d* and *S* = 0 has been considered. Recall the definition of sets $\boldsymbol E_{\mathcal {I}}(\boldsymbol {u})$ and ***E***(***u***) introduced in Eq. 1 for any non-empty $\mathcal {I}\subset \{1,\ldots , d\}$ such that $|\mathcal {I}|=k \le d$. With this notation we have

$$ \psi_k(S, T,\boldsymbol{u})=\mathbb{P} \left\{ \exists{t\in[S,T]}:\boldsymbol{W}(t)-\boldsymbol{c} t\in\boldsymbol E(\boldsymbol{u}) \right \} = \mathbb{P} \left\{ \tau_k(\boldsymbol{u})\leq T \right \} , $$

where *τ*_*k*_(***u***) is the ruin time defined by

$$\tau_k(\boldsymbol{u})=\inf\{ {t \ge S}: \boldsymbol{W}(t)-\boldsymbol{c} t\in\boldsymbol E(\boldsymbol{u})\}. $$

For the lower bound, we note that

$$\psi_k(S, T,\boldsymbol{u})=\mathbb{P} \left\{ \exists{t\in[S,T]}:\boldsymbol{W}(t)-\boldsymbol{c} t\in\boldsymbol E(\boldsymbol{u}) \right \} \ge \mathbb{P} \left\{ \boldsymbol{W}(T)-\boldsymbol{c} T\in\boldsymbol E(\boldsymbol{u}) \right \} . $$

By the fact that Brownian motion has continuous sample paths

17 $$ \boldsymbol{W}(\tau_k(\boldsymbol{u}))-\boldsymbol{c} \tau_k(\boldsymbol{u})\in\partial\boldsymbol E(\boldsymbol{u}) $$

almost surely, where *∂A* stands for the topological boundary (frontier) of the set $A \subset \mathbb {R}^{d}$. Consequently, by the strong Markov property of the Brownian motion, we can write further

$$ \begin{array}{@{}rcl@{}} \lefteqn{\mathbb{P} \left\{ \boldsymbol{W}(T) -{\boldsymbol{c}}T \in\boldsymbol E(\boldsymbol{u}) \right \} }\\ &= &{{\int}_{0}^{T}}{\int}_{\partial\boldsymbol E(\boldsymbol{u})}\mathbb{P} \left\{ \boldsymbol{W}(t)-\boldsymbol{c} t\in\mathrm{d}\boldsymbol{x}|\tau_k(\boldsymbol{u})=t \right \} \mathbb{P} \left\{ \boldsymbol{W}(T)-\boldsymbol{c}{T}\in\boldsymbol E(\boldsymbol{u})|\boldsymbol{W}(t)-\boldsymbol{c} t=\boldsymbol{x} \right \} \mathbb{P} \left\{ \tau_k(\boldsymbol{u})\in\mathrm{d} t \right \}. \end{array} $$

Crucial is that the boundary *∂****E***(***u***) can be represented as the following union

$$ \partial\boldsymbol E(\boldsymbol{u})=\underset{\underset{|\mathcal{I}|=k}{\mathcal{I}\subset\{1,\ldots, d\}}}{\bigcup}\left( \partial\boldsymbol E_{\mathcal{I}}(\boldsymbol{u})\cap\partial\boldsymbol E(\boldsymbol{u})\right)=:\underset{\underset{|\mathcal{I}|=k}{\mathcal{I}\subset\{1,\ldots, d\}}}{\bigcup} F_{\mathcal{I}}({\boldsymbol{u}}). $$

For every $\boldsymbol {x}\in F_{\mathcal {I}}(\boldsymbol {u})$ using the self-similarity of Brownian motion for all non-empty index sets $\mathcal {I} \subset \{1 ,\ldots , d\}$ and all *t* ∈ (*S*, *T*)

$$ \begin{array}{@{}rcl@{}} \mathbb{P} \left\{ \boldsymbol{W}(T)-\boldsymbol cT\in\boldsymbol E(\boldsymbol{u})|\boldsymbol{W}(t)-\boldsymbol{c} t=\boldsymbol{x} \right \} &\geq&\mathbb{P} \left\{ \boldsymbol{W}(T)-\boldsymbol cT\in\boldsymbol E_{\mathcal{I}}(\boldsymbol{u})|\boldsymbol{W}(t)-\boldsymbol{c} t=\boldsymbol{x} \right \} \\ &=&\mathbb{P} \left\{ \boldsymbol{W}_{\mathcal{I}}(T)-\boldsymbol{c}_{\mathcal{I}} T\geq \boldsymbol{u}_{\mathcal{I}}|\boldsymbol{W}(t)-\boldsymbol{c} t=\boldsymbol{x} \right \} \\ &\geq&\mathbb{P} \left\{ \boldsymbol{W}_{\mathcal{I}}(T-t)-\boldsymbol{c}_{\mathcal{I}}(T-t)\geq \boldsymbol 0 \right \} \\ &\geq&\mathbb{P} \left\{ \boldsymbol{W}_{\mathcal{I}}(T-t)\geq \boldsymbol{c}_{\mathcal{I}}(T-t) \right \} \\ &=&\mathbb{P} \left\{ \boldsymbol{W}_{\mathcal{I}}(1)\geq \boldsymbol{c}_{\mathcal{I}}\sqrt{T-t} \right \} \\ &\geq&\mathbb{P} \left\{ \boldsymbol{W}_{\mathcal{I}}(1)\geq \tilde{\boldsymbol{c}}_{\mathcal{I}}\sqrt{T} \right \} \\ &=&\mathbb{P} \left\{ \boldsymbol{W}_{\mathcal{I}}(T)\geq \tilde{\boldsymbol{c}}_{\mathcal{I}} T \right \} \\ &\geq&\underset{\underset{{|\mathcal{I}|=k}}{\mathcal{I}\subset\{1,\ldots, d\}}}{\min}\mathbb{P} \left\{ \boldsymbol{W}_{\mathcal{I}}(T)\geq \tilde{\boldsymbol{c}}_{\mathcal{I}} T \right \} , \end{array} $$

where $\tilde {c}_{i}=\max \limits (0,c_{i})$, hence for all ***x*** ∈ *∂****E***(***u***)

$$ \mathbb{P} \left\{ \boldsymbol{W}({T})-\boldsymbol{c}{T}\in\boldsymbol E(\boldsymbol{u})|\boldsymbol{W}(t)-\boldsymbol{c} t=\boldsymbol{x} \right \} \geq \underset{\underset{|\mathcal{I}|=k}{\mathcal{I}\subset\{1,\ldots, d\}}}{\min}\mathbb{P} \left\{ \boldsymbol{W}_{\mathcal{I}}({T})\geq \tilde{\boldsymbol{c}}_{\mathcal{I}} {T} \right \} .$$

Consequently, using further Eq. 17 we obtain

$$ \begin{array}{@{}rcl@{}} & &{\mathbb{P} \left\{ \boldsymbol{W}({T}) -{\boldsymbol{c}}{T} \in\boldsymbol E(\boldsymbol{u}) \right \} }\\ & &\qquad{\ge}\underset{\underset{|\mathcal{I}|=k}{\mathcal{I}\subset\{1,\ldots, d\}}}{\min}\mathbb{P} \left\{ \boldsymbol{W}_{\mathcal{I}}(T)\geq \tilde{\boldsymbol{c}}_{\mathcal{I}} T \right \} {\int}_{{S}}^{T}{\int}_{\partial\boldsymbol E(u)}\mathbb{P} \left\{ \boldsymbol{W}(t)-\boldsymbol{c} t\in\mathrm{d}\boldsymbol{x}|\tau_k(\boldsymbol{u})=t \right \} {\mathbb{P} \left\{ \tau_k(\boldsymbol{u})\in\mathrm{d} t \right \} }\\ & &\qquad=\underset{\underset{|\mathcal{I}|=k}{\mathcal{I}\subset\{1,\ldots, d\}}}{\min}\mathbb{P} \left\{ \boldsymbol{W}_{\mathcal{I}}(T)\geq \tilde{\boldsymbol{c}}_{\mathcal{I}} T \right \} \psi_k({S},T,\boldsymbol{u}) \end{array} $$

establishing the proof.

### Proof of Theorem 2.1

The results in this section hold under the assumption that Σ = ΓΓ^⊤^ is positive definite, which is equivalent with our assumption that Γ is non-singular. The next lemma is a consequence of Hashorva (2019)[Lem 2]. We recall that *φ* denotes the probability density function of Γ***B***(1).

#### **Lemma 4.1**

For any $\boldsymbol {a}\in \mathbb {R}^{d} \setminus (-\infty , 0]^{d}$ we have for some positive constants *C*_1_, *C*_2_

$$\mathbb{P} \left\{ \boldsymbol{W}(1) {- \boldsymbol{c} }>\boldsymbol {a} u \right \} \sim C_1 \mathbb{P} \left\{ \forall_{i\in I}: W_i(1) {- c_i }> a_i u \right \} \sim C_2 u^{-\alpha}\varphi(\tilde{\boldsymbol {a}}u {+ \boldsymbol{c} }), \quad u \to \infty, $$

where *α* is some integer and $\tilde {\boldsymbol {a}}$ is the solution of quadratic programming problem ${\Pi }_{\Sigma }(\boldsymbol {a}), {\Sigma }={\Gamma } {\Gamma }^{\top } $ and *I* is the unique index set that determines the solution of π_Σ_(***a***).

We agree in the following that if $\mathcal {I}$ is empty, then simply the term $A_{\mathcal {I}}(t)$ should be deleted from the expressions below; recall that $A_{\mathcal {I}}(t)$ is defined in Eq. 3.

We state next three lemmas utilised in the case $T< \infty $. Their proofs are displayed Appendix.

#### **Lemma 4.2**

Let $\mathcal {I},\mathcal {J}\subset \{1,\ldots , d\}$ be two index sets such that $\mathcal {I}\not =\mathcal {J}$ and $|\mathcal {I}|=|\mathcal {J}|=k {\ge }1$. If $\boldsymbol {a}_{\mathcal {I}\cup \mathcal {J}}$ has at least two positive components, then for any *s*, *t* ∈ [0,1] there exists some *ν* = *ν*(*s*, *t*) > 0 such that as $u\to \infty $

18 $$ \mathbb{P} \left\{ A_{\mathcal{I}}(t)\cap A_{\mathcal{J}}(s) \right \} ={o\left( e^{-\nu u^2}\right)}\underset{\underset{|\mathcal{I}^{*}|=k}{\mathcal{I}^{*} \subset\{1,\ldots, d\}}}{\sum}\mathbb{P} \left\{ A_{\mathcal{I}^{*}}(1) \right \} , $$

and

19 $$ \mathbb{P} \left\{ A_{\mathcal{I}\setminus\mathcal{J}}(t), A_{{\mathcal{J}\setminus\mathcal{I}}}(s), A_{\mathcal{I}\cap\mathcal{J}}(\min(t,s)) \right \} ={o\left( e^{-\nu u^2}\right)}\underset{\underset{|\mathcal{I}^{*}|=k}{\mathcal{I}^{*}\subset\{1,\ldots, d\}}}{\sum}\mathbb{P} \left\{ A_{\mathcal{I}^{*}}(1) \right \} . $$

#### **Lemma 4.3**

Let *S* > 0, *k* ≤ *d* be a positive integer and let $\boldsymbol {a} \in \mathbb {R}^{d}$ be given. If $\mathcal {I},\mathcal {J}\subset \{1,\ldots , d\}$ are two different index sets with *k* ≥ 1 elements such that $\boldsymbol {a}_{\mathcal {I}\cup \mathcal {J}}$ has at least one positive component, then there exist *s*_1_, *s*_2_ ∈ [*S*,1] and some positive constant *τ* such that as $u\to \infty $

20 $$ \mathbb{P} \left\{ \exists s,t\in[S,1]: A_{\mathcal{I}}(s)\cap A_{\mathcal{J}}(t) \right \} = {o\left( e^{\tau u}\right)} \mathbb{P} \left\{ A_{\mathcal{I}\setminus\mathcal{J}}(s_1)\cap A_{\mathcal{J}\setminus\mathcal{I}}(s_2)\cap A_{\mathcal{I}\cap\mathcal{J}}(\min(s_1,s_2)) \right \} . $$

#### Case $T< \infty $

According to Theorem 1.1 and Lemma 4.1 it is enough to show the proof for *S* ∈ (0, *T*). In view of the self-similarity of Brownian motion we assume for simplicity *T* = 1. Recall that in our notation Σ = ΓΓ^⊤^ is the covariance matrix of ***W***(1) which is non-singular and we denote its pdf by *φ*. In view of Eqs. 19 and 20 for all *S* ∈ (0,1) there exists some *ν* > 0 such that as $u\to \infty $

$$\underset{\underset{| \mathcal{I}|=|\mathcal{J}|=k, \mathcal{I}\not= \mathcal{J}}{\mathcal{I},\mathcal{J} \subset\{1,\ldots, d\}}}{\sum}\mathbb{P} \left\{ \exists s,t\in[S,1]: A_{\mathcal{I}}(s)\cap A_{\mathcal{J}}(t) \right \} ={o\left( e^{- \nu u^2} \right)}\underset{\underset{|\mathcal{I}|=k}{\mathcal{I}\subset\{1,\ldots, d\}}}{\sum}\mathbb{P} \left\{ A_{\mathcal{I}}(1) \right \} . $$

Note that we may utilise Eqs. 19 and 20 for sets $\mathcal {I}$ and $\mathcal {J}$ of length *k*, because of the assumption that ***a*** has no more than *k* − 1 non-positive components. Hence any vector $\boldsymbol {a}_{\mathcal {I}}$ has at least one positive component.

Further, by Theorem 1.1 and the inclusion-exclusion formula we have that for some *K* > 0 and all *u* sufficiently large

$$ \psi_{k}(S, 1,\boldsymbol{u}) \le {K}{\sum}_{\substack{I\subset\{1,\ldots, d\}\\|I|=k}}\mathbb{P} \left\{ A_{\mathcal{I}}(1) \right \} . $$

Hence the claim follows from Eqs. 4 and 5.

#### Case $T=\infty $

Using the self-similarity of Brownian motion we have

$$ \begin{array}{@{}rcl@{}}\mathbb{P} \left\{ \exists t>0: A_{\mathcal{I}}(t) \right \} =\mathbb{P} \left\{ \exists t>0:\boldsymbol{W}_{\mathcal{I}}(ut)\ge(\boldsymbol {a}+\boldsymbol{c} t)_{{\mathcal{I}}} u \right \} &=&\mathbb{P} \left\{ \exists t>0:\boldsymbol{W}_{\mathcal{I}}(t)\ge (\boldsymbol {a}+\boldsymbol{c} t)_{{\mathcal{I}}}\sqrt{u} \right \} \\ &=&\mathbb{P} \left\{ \exists t>0:A^{*}_{\mathcal{I}}(t) \right \} , \end{array} $$

where

21 $$ A^{*}_{\mathcal{I}}(t)= \{ \boldsymbol{W}_{\mathcal{I}}(t)\ge (\boldsymbol {a}+\boldsymbol{c} t)_{{\mathcal{I}}}\sqrt{u} \} . $$

For *t* > 0 define

22 $$ r_{\mathcal{I}}(t)=\min_{\boldsymbol{x}\ge\boldsymbol {a}_{\mathcal{I}}+\boldsymbol{c}_{\mathcal{I}} t}\frac{1}{t}\boldsymbol{x}^{\top}{\Sigma}^{-1}_{\mathcal{I}\mathcal{I}}\boldsymbol{x}, \ \ {\Sigma}_{\mathcal{I}\mathcal{I}}=Var(\boldsymbol{W}_{\mathcal{I}}(1)), \ \ {\Sigma}^{-1}_{\mathcal{I}\mathcal{I}}= ({\Sigma}_{\mathcal{I}\mathcal{I}})^{-1}. $$

Since $\lim _{t\downarrow 0}r_{{\mathcal {I}}}(t)=\infty $ we set below $r_{{\mathcal {I}}}(0)=\infty $.

In view of Lemma 4.1 we have as $u\to \infty $

$$ \begin{array}{@{}rcl@{}}\mathbb{P} \left\{ A_{\mathcal{I}}^{*}(t) \right \} \sim C_1 u^{-\alpha/2}\varphi_{\mathcal{I},t}((\widetilde{\boldsymbol {a}_{\mathcal{I}}+\boldsymbol{c}_{\mathcal{I}} t})\sqrt{u})= C_2 u^{{-\alpha/2}} e^{-\frac{r_{\mathcal{I}}(t) u}{2}}, \end{array} $$

where $\widetilde {\boldsymbol {a}_{\mathcal {I}}+\boldsymbol {c}_{\mathcal {I}} t}$ is the solution of quadratic programming problem ${\Pi }_{t{\Sigma }_{\mathcal {I}\mathcal {I}}}(\boldsymbol {a}_{\mathcal {I}}+\boldsymbol {c}_{\mathcal {I}} t)$ and $\varphi _{\mathcal {I},t}(\boldsymbol {x})$ is the pdf of $\boldsymbol {W}_{\mathcal {I}}(t)$, *α* is some integer and *C*_1_, *C*_2_ are positive constant that do not depend on *u*. For notational simplicity we shall omit below the subscript $\mathcal {I}$.

The rest of the proof is established by utilising the following lemmas, whose proofs are displayed in Appendix.

#### **Lemma 4.4**

Let *k* ≤ *d* be a positive integer and let $\boldsymbol {a},\boldsymbol {c}\in \mathbb {R}^{d}$. Consider two different sets $\mathcal {I},\mathcal {J}\subset \{1,{\ldots } ,d\}$ of cardinality *k*. If both $\boldsymbol {a}_{\mathcal {I}}+\boldsymbol {c}_{\mathcal {I}} t$ and $\boldsymbol {a}_{\mathcal {J}}+\boldsymbol {c}_{\mathcal {J}} t$ have at least one positive component for all *t* > 0 and both $\boldsymbol {c}_{\mathcal {I}}$ and $\boldsymbol {c}_{\mathcal {J}}$ also have at least one positive component, then in case $\hat {t}_{\mathcal {I}}:=\arg \min \limits _{t>0}~r_{\mathcal {I}}(t)\not =\hat {t}_{\mathcal {J}}:=\arg \min \limits _{t>0}~r_{\mathcal {J}}(t)$,

$$\mathbb{P} \left\{ \exists s,t>0:~A^{*}_{\mathcal{I}}(t){\cap} A^{*}_{\mathcal{J}}(s) \right \} =o(\mathbb{P} \left\{ A^{*}_{\mathcal{I}}(\hat{t}_{\mathcal{I}}) \right \} +\mathbb{P} \left\{ A^{*}_{\mathcal{J}}(\hat{t}_{\mathcal{J}}) \right \} ), \quad u\to \infty. $$

#### **Lemma 4.5**

Under the settings of Lemma 4.4, if ***a*** + ***c****t* has no more than *k* − 1 non-positive component for all *t* > 0 and ***c*** has no more than *k* − 1 non-positive components, then in case $\hat {t}_{\mathcal {I}}:=\arg \min \limits _{t>0}~r_{\mathcal {I}}(t)=\hat {t}_{\mathcal {J}}:=\arg \min \limits _{t>0}~r_{\mathcal {J}}(t)$

$$ \mathbb{P} \left\{ \exists s,t>0:~A^{*}_{\mathcal{I}}(t){\cap} A^{*}_{\mathcal{J}}(s) \right \} =o\left( \underset{\underset{|\mathcal{K}|=k}{\mathcal{K}\subset\{1{\ldots} d\}}}{\sum}\mathbb{P} \left\{ A^{*}_{\mathcal{K}}(\hat{t}_{\mathcal{K}}) \right \} \right), \quad u\to \infty. $$

Combining the above two lemmas we have that for any two index sets $\mathcal {I},\mathcal {J}\subset \{1,\ldots , d\}$ of cardinality *k*, there is some index set $\mathcal {K}\subset \{1,\ldots , d\}$ such that as $u\to \infty $

$$ \begin{array}{@{}rcl@{}}\mathbb{P} \left\{ \exists s,t>0: A_{\mathcal{I}}^{*}(s){\cap} A_{\mathcal{J}}^{*}(t) \right \} =o\left( \mathbb{P} \left\{ \exists t>0:A_{\mathcal{K}}^{*}(t) \right \} \right), \end{array} $$

which is equivalent with

$$ \begin{array}{@{}rcl@{}}\mathbb{P} \left\{ \exists s,t>0: A_{\mathcal{I}}(s){\cap} A_{\mathcal{J}}(t) \right \} =o\left( \mathbb{P} \left\{ \exists t>0:A_{\mathcal{K}}(t) \right \} \right). \end{array} $$

The proof follows now by Eqs. 4 and 5.

### Proof of Theorem 2.3

Below we set

$$ \delta(u,{\Lambda}):=1-{\Lambda}u^{-2}$$

and denote by $\tilde { \boldsymbol {a}}$ the unique solution of the quadratic programming problem π_Σ_(***a***).

We denote below by *I* the index set that determines the unique solution of π_Σ_(***a***), where $\boldsymbol {a} \in \mathbb {R}^{d}$ has at least one positive component (see Lemma 2.2). If *J* = {1, … , *d*}∖ *I* is non-empty, then we set below $U=\{j\in J:\tilde { a}_{j}= a_{j}\}$. The number of elements |*I*| of *I* is denoted by *m*, which is a positive integer.

The next lemma is proved in Appendix.

#### **Lemma 4.6**

For any Λ > 0, $\boldsymbol {a}\in \mathbb {R}^{d} \setminus (-\infty ,0]^{d}, {\boldsymbol {c} \in \mathbb {R}^{d}}$ and all sufficiently large u there exist *C* > 0 such that

23 $$ m(u,{\Lambda}):=\mathbb{P} \left\{ \exists_{t\in[0,\delta(u,{\Lambda})]}:\boldsymbol{W}(t)-t\boldsymbol{c}> u\boldsymbol {a} \right \} \le e^{-{\Lambda}/C}\frac{\mathbb{P} \left\{ \boldsymbol{W}(1)\ge \boldsymbol {a} u+\boldsymbol{c} \right \} }{\mathbb{P} \left\{ \boldsymbol{W}(1)>\max(\boldsymbol{c},0) \right \} } $$

and further

24 $$ M(u,{\Lambda}):=\mathbb{P} \left\{ \exists_{t\in[\delta(u,{\Lambda}),1]}:\boldsymbol{W}(t)-t\boldsymbol{c} >u\boldsymbol {a} \right \} \sim C(\boldsymbol{c})K([0,{\Lambda}])u^{-m}\varphi(u\tilde{\boldsymbol {a}}+\boldsymbol{c}), $$

where $C(\boldsymbol {c})= \mathbb {P} \left \{ \boldsymbol {W}_{U}(1)> \boldsymbol {c}_{U} \lvert \boldsymbol {W}_{I}(1)> \boldsymbol {c}_{I} \right \} $ and for $\boldsymbol \lambda = {\Sigma }^{-1} \tilde {\boldsymbol {a}}$

$$ \begin{array}{@{}rcl@{}}E([{{\Lambda}_1},{{\Lambda}_2}])={\int}_{\mathbb{R}^{m}} \mathbb{P} \left\{ \exists_{t\in[{{\Lambda}_1},{{\Lambda}_2}]}:\boldsymbol{W}_{I}(t)-t\boldsymbol {a}_{I}>\boldsymbol{x}_I \right \} e^{\boldsymbol\lambda_I^{\top} \boldsymbol{x}_I}\mathrm{d}\boldsymbol{x}_I \in (0,\infty) \end{array} $$

for all constants Λ_1_ < Λ_2_. We set *C*(***c***) equal 1 if *U* defined in Remark 2.4 is empty. Further we have

25 $$ \lim\limits_{{\Lambda}\to\infty}E([0,{\Lambda}])={\int}_{\mathbb{R}^{m}}\mathbb{P} \left\{ \exists_{t\geq 0}:\boldsymbol{W}_{I}(t)-t\boldsymbol {a}_{I}>\boldsymbol{x}_I \right \} e^{\boldsymbol \lambda_I^{\top} \boldsymbol{x}_I}\mathrm{d}\boldsymbol{x}_I\in(0,\infty). $$

First note that for all Λ, *u* positive

$$M(u,{\Lambda})\leq\mathbb{P} \left\{ \exists_{t\in[0,1]}:\boldsymbol{W}(t)-t\boldsymbol{c}>u\boldsymbol {a} \right \} \leq M(u,{\Lambda})+m(u,{\Lambda}). $$

In view of Lemmas 4.6 and 4.1

$$ \begin{array}{@{}rcl@{}}\lim_{{\Lambda}\to\infty}\lim_{u\to\infty}\frac{m(u,{\Lambda})}{M(u,{\Lambda})}=0, \end{array} $$

hence

$$ \begin{array}{@{}rcl@{}} \lim_{{\Lambda}\to\infty}\lim_{u\to\infty}\frac{\mathbb{P} \left\{ \exists_{t\in[0,1]}:\boldsymbol{W}(t)-t\boldsymbol{c}>u\boldsymbol {a} \right \} }{M(u,{\Lambda})}=1 \end{array} $$

and thus the proof follows applying Eq. 24.

### Proof of Eq. 14

The proof is similar to that of Dȩbicki et al. (2017)[Thm 2.5] and therefore we highlight only the main steps. If *T* > *S* ≥ 0 by the definition of *τ*(*u*) and the self-similarity of Brownian motion

$$ \begin{array}{@{}rcl@{}}\frac{\tau(u)}{T}=\inf\{t\ge 0:\boldsymbol{W}(Tt)-tT\boldsymbol{c}>\boldsymbol {a} u\}=\inf\{t\ge 0:\boldsymbol{W}(t)-t\sqrt{T}\boldsymbol{c}>\boldsymbol {a} u/\sqrt{T}\}. \end{array} $$

Thus, without loss of generality in the rest of the proof we suppose that *T* = 1 > *S* ≥ 0.

We note that

$$ \begin{array}{@{}rcl@{}} &&\mathbb{P} \left\{ u^2(1-\tau(u))\geq x| \tau(u)\in [S,1] \right \} =\frac{\mathbb{P} \left\{ u^2(1-\tau(u))\geq x, \tau(u)\in [S,1] \right \} }{\mathbb{P} \left\{ \tau(u)\in [S,1] \right \} }\\ &&\qquad= \frac{\mathbb{P} \left\{ u^2(1-\tau(u))\geq x, \tau(u)\leq 1 \right \} }{\mathbb{P} \left\{ \tau(u)\in [S,1] \right \} } -\frac{\mathbb{P} \left\{ u^2(1-\tau(u))\geq x, \tau(u)\leq S \right \} }{\mathbb{P} \left\{ \tau(u)\in [S,1] \right \} }\\ &&\qquad=P_1(u)-P_2(u). \end{array} $$

Next, for $\tilde {x}(u)=1-\frac {x}{u^{2}}$

$$ \begin{array}{@{}rcl@{}} P_{1}(u)&=&\frac{\mathbb{P} \left\{ \tau(u)\leq \tilde{x}(u) \right \} }{\mathbb{P} \left\{ \tau(u)\in [S,1] \right \} }\sim \frac{\mathbb{P} \left\{ \exists_{t\in[0,\tilde{x}(u)]}:\boldsymbol{W}(t)-\boldsymbol{c} t>u\boldsymbol {a} \right \} } {\mathbb{P} \left\{ \exists_{t\in[0,1]}:\boldsymbol{W}(t)-\boldsymbol{c} t>u\boldsymbol {a} \right \} }\\ &=& \frac{\mathbb{P} \left\{ \exists_{t\in[0,1]}:\boldsymbol{W}(t)-(\boldsymbol{c}\sqrt{\tilde{x}(u)}) t>\frac{u}{\sqrt{\tilde{x}(u)}}\boldsymbol {a} \right \} } {\mathbb{P} \left\{ \exists_{t\in[0,1]}:\boldsymbol{W}(t)-\boldsymbol{c} t>u\boldsymbol {a} \right \} }, \ u \to \infty. \end{array} $$

Hence by Theorem 2.3, the fact that

$$ \varphi\left( \frac{u}{\sqrt{\tilde{x}(u)}}\tilde{\boldsymbol {a}}+(\boldsymbol{c}\sqrt{\tilde{x}(u)})\right)=\varphi(u\tilde{\boldsymbol {a}}+\boldsymbol{c})e^{-\frac{1}{2}\left( \frac{1}{\tilde{x}(u)}-1\right)u^2\tilde{\boldsymbol {a}}^{\top}{\Sigma}^{-1}\tilde{\boldsymbol {a}}}e^{-\frac{1}{2}(\tilde{x}(u)-1)\boldsymbol{c}^{\top}{\Sigma}^{-1}\boldsymbol{c}} $$

and

$$ \lim_{u \to \infty} e^{-\frac{1}{2}\left( \frac{1}{\tilde{x}(u)}-1\right)u^2\tilde{\boldsymbol {a}}^{\top}{\Sigma}^{-1}\tilde{\boldsymbol {a}}}= e^{-x\frac{\tilde{\boldsymbol {a}}^{\top}{\Sigma}^{-1}\tilde{\boldsymbol {a}}}{2}},\quad \lim_{u \to \infty}e^{-\frac{1}{2}(\tilde{x}(u)-1)\boldsymbol{c}^{\top}{\Sigma}^{-1}\boldsymbol{c}}= 1 $$

we obtain

26 $$ \lim_{u\to\infty} P_{1}(u)= e^{-x\frac{\tilde{\boldsymbol {a}}^{\top}{\Sigma}^{-1}\tilde{\boldsymbol {a}}}{2}}. $$

Moreover, following the same reasoning as above

27 $$ P_{2}(u)=\frac{\mathbb{P} \left\{ \tau(u)\leq S \right \} }{\mathbb{P} \left\{ \tau(u)\in [S,1] \right \} } \sim \frac{\mathbb{P} \left\{ \tau(u)\leq S \right \} }{\mathbb{P} \left\{ \tau(u)\leq 1 \right \} }\to 0 $$

as $u\to \infty $. Thus, combination of Eqs. 26 with 27 leads to

$$ \lim_{u \to \infty} \mathbb{P} \left\{ u^2(1-\tau(u))\geq x|\tau(u) \in [S,1] \right \} = e^{-x\frac{\tilde{\boldsymbol {a}}^{\top}{\Sigma}^{-1}\tilde{\boldsymbol {a}}}{2}}. $$

## Appendix

### **Lemma A.1**

If for $\boldsymbol {a}\in (\mathbb {R}\cup \{-\infty \})^{d}$ and $\mathcal {I}\subset \{1,\ldots , d\}$ such that $\boldsymbol {a}_{\mathcal {I}}$ has at least two positive components and Γ is non-singular, then for all *t* > 0

$$ \begin{array}{@{}rcl@{}} \mathbb{P} \left\{ A_{\mathcal{I}}(t) \right \} ={o\left( e^{-\nu u^2}\right)}{\sum}_{i\in \mathcal{I}}\mathbb{P} \left\{ A_{\mathcal{I}\setminus\{i\}}(t) \right \} , \quad u\to \infty, \end{array} $$

where $\nu =\nu (t,\mathcal {I})>0$ does not depend on *u*.

### *Remark A.2*

Lemma A.1 implies that for any vector $\boldsymbol {a}\in (\mathbb {R}\cup \{-\infty \})^{d}$ and for any *d*-dimensional Gaussian random vector ***W***, if ***a*** has at least two positive components, there exists some positive constant *η* and *i* ∈{1…*d*} such that as $u\to \infty $

$$ \begin{array}{@{}rcl@{}} \mathbb{P} \left\{ \boldsymbol{W}>\boldsymbol {a} u \right \} =o(e^{-\eta u^2})\mathbb{P} \left\{ \boldsymbol{W}_{K}>\boldsymbol {a}_{K}u \right \} , \quad K= \{1,\ldots, d\}\setminus \{i\} . \end{array} $$

### *Proof of Lemma A.1*

For notational simplicity we shall assume that $\mathcal {I}= \{1,\ldots , d \}$ and set $K_{i}=\mathcal {I}\setminus \{i\} $. By the assumption for all $i\in \mathcal {I}$ the vector $\boldsymbol {a}_{K_{i}}$ has at least one positive component and Σ = ΓΓ^⊤^ is positive definite. In view of Lemma 4.1 for any fixed *t* > 0 and some *C*_1_, *C*_2_ two positive constants we have

$$\mathbb{P} \left\{ A_{\mathcal{I}}(t) \right \} \sim C_1 u^{\alpha_1}\varphi_t(\tilde{\boldsymbol {a}}u+\boldsymbol{c}), \quad \mathbb{P} \left\{ A_{K_i}(t) \right \} \sim C_2 u^{\alpha_2}\varphi_t(\bar{\boldsymbol {a}_i}u +\boldsymbol{c}), \quad u\to \infty, $$

where *φ*_*t*_ is the pdf of ***W***(*t*) with covariance matrix Σ(*t*) = *t*Σ and $\tilde {\boldsymbol {a}}=\arg \min \limits _{\boldsymbol {x} \ge \boldsymbol {a}} \boldsymbol {x}^{\top } {\Sigma }^{-1}(t)\boldsymbol {x}$, $\bar {\boldsymbol {a}_{i}}=\arg \min \limits _{\boldsymbol {x}\in S_{i}} \boldsymbol {x}^{\top }{\Sigma }^{-1}(t)\boldsymbol {x},$ with $S_{i}=\{\boldsymbol {x}\in \mathbb {R}^{d}:~\forall j \in K_{i} : x_{j}\geq a_{j}\}$. Since $\{\boldsymbol {x}\in \mathbb {R}^{d}: \boldsymbol {x} \ge \boldsymbol {a} \}\subset S_{i}$, then clearly

$$\tilde{\boldsymbol {a}}^{\top} {\Sigma}^{-1}(t) \tilde{\boldsymbol {a}} \geq\bar{\boldsymbol {a}_{i}}^{\top} {\Sigma}^{-1}(t)\bar{\boldsymbol {a}_{i}}$$

for any *i* ≤ *d*. Next, if we have strict inequality for some *i* ≤ *d*, i.e., $\tilde {\boldsymbol {a}}^{\top }{\Sigma }^{-1}(t) \tilde {\boldsymbol {a}}>\bar {\boldsymbol {a}_{i}}^{\top } {\Sigma }^{-1}(t)\bar {\boldsymbol {a}_{i}}$, then it follows that

$$\mathbb{P} \left\{ {A_{\mathcal{I}}(t)} \right \}\sim C u^{\alpha_1}\varphi_t(\tilde{\boldsymbol {a}}u +\boldsymbol{c})=o\left( e^{-\nu u^2}\mathbb{P} \left\{ {A_{K_i}(t)} \right \}\right), \quad u\to \infty $$

for $\nu =\frac {1}{2}\left (\tilde {\boldsymbol {a}}^{\top }{\Sigma }^{-1}(t) \tilde {\boldsymbol {a}}-\bar {\boldsymbol {a}_{i}}^{\top } {\Sigma }^{-1}(t)\bar {\boldsymbol {a}_{i}}\right ) >0$, hence the claim follows.

Let us consider now the extreme case that for all *i* ≤ *d* we have $\tilde {\boldsymbol {a}}^{\top }{\Sigma }^{-1}\tilde {\boldsymbol {a}}=\bar {\boldsymbol {a}_{i}}^{\top } {\Sigma }^{-1}\bar {\boldsymbol {a}_{i}}$. Define the following set

$$E=\{\boldsymbol{x}\in \mathbb{R}^d:\boldsymbol{x}^{\top}{\Sigma}^{-1}(t)\boldsymbol{x}\leq\tilde{\boldsymbol {a}}^{\top} {\Sigma}^{-1}(t) \tilde{\boldsymbol {a}}\}. $$

Since Σ(*t*) is positive definite, *E* is a full dimensional ellipsoid in $\mathbb {R}^{d}$. By the definition, $E\cap S_{i}=\{\tilde {\boldsymbol {a}}\}$. Define the following lines in $\mathbb {R}^{d}$

$$ \begin{array}{@{}rcl@{}} l_i=\{\boldsymbol{x}\in \mathbb{R}^d:\forall i\in K_i, x_i=\tilde{\textbf{a}}_i\} \end{array} $$

and observe that since *l*_*i*_ ∈ *S*_*i*_, then $l_{i}\cap E=\{\tilde {\boldsymbol {a}}\}$, and they are linearly independent. Since the boundary of *E* is smooth, there can not be more than *d* − 1 linearly independent tangent lines at the point $\tilde {\boldsymbol {a}}$, which leads to a contradiction. □

### *Proof of Lemma 4.2*

First note that since $\mathcal {I}\not = \mathcal {J}$, then $|\mathcal {I}\cup \mathcal {J}|\geq k+1$. Consequently, we can find some index set $\mathcal {K}$ such that

$$|\mathcal{K}|=k+1, \quad \mathcal{K}\subset \mathcal{I}\cup \mathcal{J}$$

and further $ \boldsymbol {a}_{\mathcal {K}}$ has at least two positive components. Applying Lemma A.1 for any *t* ∈ [0, 1] and some *ν* > 0

$$ \begin{array}{@{}rcl@{}}\mathbb{P} \left\{ A_{\mathcal{K}}(t) \right \} = o\left( e^{-\nu u^2}\right){\sum}_{j\in \mathcal{K}}\mathbb{P} \left\{ A_{\mathcal{K}\setminus \{j\}}(t) \right \} , \quad u\to \infty. \end{array} $$

If *s* = *t*, then applying Lemma 4.1

$$0\leq\mathbb{P} \left\{ A_{\mathcal{I}}(t)\cap A_{\mathcal{J}}(t) \right \} =\mathbb{P} \left\{ A_{\mathcal{I}\cup \mathcal{J}}(t) \right \} \leq\mathbb{P} \left\{ A_{\mathcal{K}}(t) \right \} = o\left( e^{- \nu u^2} \right){\sum}_{\substack{\mathcal{I}^{*}\subset\{1,\ldots, d\}\\ \mathcal{I}^{*}|=k}}\mathbb{P} \left\{ A_{\mathcal{I}}^{*}(t) \right \} . $$

Next, if *s* < 1, then applying Lemma 4.1 we obtain

$$0\leq\mathbb{P} \left\{ A_{\mathcal{I}}(t)\cap A_{\mathcal{J}}(s) \right \} \leq\mathbb{P} \left\{ A_{\mathcal{J}}(s) \right \} =o\left( e^{-\nu u^2}\mathbb{P} \left\{ A_{\mathcal{J}}(1) \right \} \right)=o\left( e^{-\nu u^2}\right){\sum}_{\substack{\mathcal{I}^{*}\subset\{1,\ldots, d\}\\|\mathcal{I}^{*}|=k}}\mathbb{P} \left\{ A_{\mathcal{I}^{*}}(1) \right \} . $$

A similar asymptotic bound follows for *t* < 1, whereas if *s* = *t* = 1, the first claim follows directly from the case *s* = *t* discussed above. We show next Eq. 19. If *s* < *t*, then *s* < 1 and applying Lemma 4.1 we obtain

$$ \begin{array}{@{}rcl@{}} 0 &\le& \mathbb{P} \left\{ A_{\mathcal{I}\setminus\mathcal{J}}(t), A_{\mathcal{J}\setminus\mathcal{I}}(s), A_{\mathcal{I}\cap\mathcal{J}}(\min(t,s)) \right \} \\ &\leq &\mathbb{P} \left\{ A_{\mathcal{J}}(s) \right \} =o\left( e^{-\nu u^2}\mathbb{P} \left\{ A_{\mathcal{J}}(1) \right \} \right) \\ &=&o\left( e^{-\nu u^2} \right){\sum}_{\substack{\mathcal{K}\subset\{1,\ldots, d\}\\|\mathcal{K}|=k}}\mathbb{P} \left\{ A_{\mathcal{K}}(1) \right \} . \end{array} $$

A similar asymptotic bound follows for *t* < *s* or *s* = *t* ≤ 1 by applying Eq. 18 establishing the proof. □

### *Proof of Lemma 4.3*

Define for *s*, *t* ∈ [*S*, 1] the Gaussian random vector

$$\mathcal{W}(s,t)=(\boldsymbol{W}_{\mathcal{I}\setminus\mathcal{J}}(s)^{\top}, \boldsymbol{W}_{\mathcal{J}\setminus\mathcal{I}}(t)^{\top}, \boldsymbol{W}_{\mathcal{I}\cap\mathcal{J}}(\min(s,t))^{\top})^{\top},$$

with covariance matrix *D*(*s*, *t*). We show first that this matrix is positive definite. For this we assume that *s* ≤ *t*. As *D*(*s*, *t*) is some covariance matrix, we know that it is non-negative definite. Choose some vector $\boldsymbol v\in \mathbb {R}^{d}$. It is sufficient to show that if ***v***^⊤^*D*(*s*, *t*)***v*** = ***0***, then ***v*** = ***0*** (here $\boldsymbol 0=(0, \ldots ,0)^{\top }\in \mathbb {R}^{d}$). Note that

$$ \boldsymbol v^{\top} D(s,t)\boldsymbol v=Var(\langle\mathcal{W}(s,t),\boldsymbol v\rangle)=Var(\langle\boldsymbol{W}(s),\boldsymbol v\rangle+\langle\boldsymbol{W}_{\mathcal{J}\setminus\mathcal{I}}(t)-\boldsymbol{W}_{\mathcal{J}\setminus\mathcal{I}}(s),\boldsymbol v_{\mathcal{J}\setminus\mathcal{I}}\rangle). $$

Using that ***W***(*t*) has independent increments, this variance is equal to the sum of the variances. Hence, both of them should be equal to zero. In particular it means that *V* *a**r*(〈***W***(*s*), ***v***〉) = 0. Hence, as $s\geqslant S>0$, we have that ***v*** = ***0***. Thus, *D*(*s*, *t*) is positive definite and *D*^− 1^(*s*, *t*) exists. Set further

$${\mathfrak{a}=(\boldsymbol {a}_{\mathcal{I}\setminus\mathcal{J}}^{\top},\boldsymbol {a}_{\mathcal{J}\setminus\mathcal{I}}^{\top}, \boldsymbol {a}_{\mathcal{I}\cap\mathcal{J}}^{\top})^{\top}}, \quad {\mathfrak{c}(s,t)=(s\boldsymbol{c}_{\mathcal{I}\setminus\mathcal{J}}^{\top}, t\boldsymbol{c}_{\mathcal{J}\setminus\mathcal{I}}^{\top}, \min(s,t)\boldsymbol{c}_{\mathcal{I}\cap\mathcal{J}}^{\top})^{\top}}.$$

With this notation we have

$$ \begin{array}{@{}rcl@{}}\mathbb{P} \left\{ \exists s,t\in[S,1]:A_{\mathcal{I}}(s)\cap A_{\mathcal{J}}(t) \right \} \leq\mathbb{P} \left\{ \exists s,t\in[S,1]:\mathcal{W}(s,t)-\mathfrak{c}(s,t)\geq\mathfrak{a}u \right \} . \end{array} $$

Let $\tilde {\mathfrak {a}}(s,t) = \arg \min \limits _{{\boldsymbol {x}}\geq \mathfrak {a}} \boldsymbol {x}^{\top } D^{-1}(s,t) \boldsymbol {x}$ be the unique solution of ${\Pi }_{D(s,t)}(\mathfrak {a})$ and let further $ \mathfrak {w}(s,t) =D^{-1}(s,t)\tilde {\mathfrak {a}}(s,t)$ be the solution of the dual problem. We denote by *I*(*s*, *t*) the index set related to the quadratic programming problem ${\Pi }_{D(s,t)}(\mathfrak {a})$. Then $ \mathfrak {w}(s,t)$ has non-negative components and according to Lemma 2.2 since both *s*, *t* ≥ *S* > 0 we have

$$\mathfrak{a}^{\top}\mathfrak{w}(s,t)=\tilde{\mathfrak{a}}^{\top}(s,t)\mathfrak{w}(s,t) =\tilde{\mathfrak{a}}^{\top}(s,t) D^{-1}(s,t) \tilde{\mathfrak{a}}(s,t)>0.$$

Consequently, we have

$$ \begin{array}{@{}rcl@{}} \mathbb{P} \left\{ \exists s,t\in[S,1]:\mathcal{W}(s,t)-\mathfrak{c}(s,t)\geq\mathfrak{a}u \right \} &\leq&\mathbb{P} \left\{ \exists s,t\in[S,1]: \mathfrak{w}^{\top}(s,t) \left( \mathcal{W}(s,t)-\mathfrak{c}(s,t)\right)\geq u \mathfrak{w}^{\top}(s,t) \tilde{\mathfrak{a}}(s,t) \right \} \\ &=&\mathbb{P} \left\{ \exists s,t\in[S,1]:\frac{\mathfrak{w}^{\top}(s,t) \left( \mathcal{W}(s,t)-\mathfrak{c}(s,t)\right)}{\mathfrak{w}^{\top}(s,t) \tilde{\mathfrak{a}}(s,t)}\geq u \right \} \\ &\leq&\mathbb{P} \left\{ \exists s,t\in[S,1]:\frac{\mathfrak{w}^{\top}(s,t) \mathcal{W}(s,t)}{\mathfrak{w}^{\top}(s,t) \tilde{\mathfrak{a}}(s,t)}\geq u+\mathfrak{C} \right \} \end{array} $$

for any positive *u*, where $\mathfrak {C}=\min \limits _{s,t\in [S,1]}\frac {\mathfrak {w}^{\top }(s,t)\mathfrak {c}(s,t)}{\mathfrak {w}^{\top }(s,t)\tilde {\mathfrak {a}}(s,t)}$. Moreover, for some *s*_1_, *s*_2_ ∈ [*S*, 1]

$$ \sigma^{2}=\sup_{s,t\in[S,1]}\mathbb{E}\left\{ \left( \frac{{\mathfrak{w}^{\top}(s,t)\mathcal{W}(s,t)}}{{\mathfrak{w}^{\top}(s,t) \tilde{\mathfrak{a}}(s,t)}}\right)^2\right\} =\sup_{s,t\in[S,1]}\frac{1}{\tilde{\mathfrak{a}}^{\top}(s,t) D^{-1}(s,t)\tilde{\mathfrak{a}}(s,t)} =\frac{1}{\tilde{\mathfrak{a}}^{\top}(s_1,s_2)D^{-1}(s_1,s_2)\tilde{\mathfrak{a}}(s_1,s_2)} $$

since [*S*, 1]^2^ is compact. Moreover, one can check that for some positive constant *G* and *s*_1_, *s*_2_, *t*_1_, *t*_2_ ∈ [*S*, 1]

28 $$ \mathbb{E}\left\{ \left( \frac{\mathfrak{w}^{\top}(s_{1},t_{1}) \mathcal{W}(s_{1},t_{1})}{\mathfrak{w}^{\top}(s_{1},t_{1}) \tilde{\mathfrak{a}}(s,t)}-\frac{\mathfrak{w}^{\top}(s_{2},t_{2}) \mathcal{W}(s_{2},t_{2})}{\mathfrak{w}^{\top}(s_{2},t_{2}) \tilde{\mathfrak{a}}(s,t)}\right)^{2}\right\}\leq G [\left\lvert s_{1}-s_{2} \right\rvert+\left\lvert t_{1}-t_{2} \right\rvert]. $$

Thus, utilizing Piterbarg inequality, see e.g., Piterbarg (1996)[Thm 8.1], we have that there exist positive constants *C*, *γ* such that

$$ \begin{array}{@{}rcl@{}}\mathbb{P} \left\{ \exists s,t\in[S,1]:\mathcal{W}(s,t)-\mathfrak{c}(s,t)\geq\mathfrak{a}u \right \} \leq Cu^{\gamma} e^{-(u+\mathfrak{C})^2/2\sigma^2} \end{array} $$

for all *u* positive. Further, by Lemma 4.1 for some constants *α*, *C*^∗^, *C*^+^ as $u\to \infty $

$$ \begin{array}{@{}rcl@{}} &&\mathbb{P} \left\{ A_{\mathcal{I}\setminus\mathcal{J}}(s_1),A_{\mathcal{J}\setminus\mathcal{I}}(s_2),A_{\mathcal{I}\cap\mathcal{J}}(\min(s_1,s_2)) \right \} \\ &&\qquad=\mathbb{P} \left\{ \mathcal{W}(s_1,s_2)-\mathfrak{c}(s_1,s_2)\geq \mathfrak{a}u \right \} \\ &&\qquad\sim C^{*}u^{-\alpha}e^{-\frac{1}{2}(\tilde{\mathfrak{a}}(s_1,s_2)u+ \mathfrak{c}(s_1,s_2))^{\top} {D^{-1}(s_1,s_2)}(\tilde{\mathfrak{a}}(s_1,s_2)u+\mathfrak{c}(s_1,s_2))}\\ &&\qquad= C^+u^{-\alpha}e^{-\frac{u^2}{2{\sigma^2}}}e^{-u(\tilde{\mathfrak{a}}_{s_1,s_2})^{\top}{D^{-1}(s_1,s_2)}(\mathfrak{c}(s_1,s_2))}. \end{array} $$

Hence the claim follows for $\tau =|\mathfrak {C}/\sigma ^{2}|+\sup _{s,t\in [S,1]}|\tilde {\mathfrak {a}}(s,t)D^{-1}(s,t)\mathfrak {c}(s,t)|+1$. □

### **Lemma A.3**

The function $r_{\mathcal {I}}(t),t>0$ defined in Eq. 22 is convex and if $\boldsymbol {c}_{\mathcal {I}}$ has at least one positive component, then there exists *T* > 0 such that for some positive *s* and any *t* > 0

29 $$ r_{\mathcal{I}}(T+t)\ge r_{\mathcal{I}}(T)+st. $$

Moreover, if $\boldsymbol {a}_{\mathcal {I}}+\boldsymbol {c}_{\mathcal {I}} t$ for any *t* > 0 have at least one positive component, then $r_{\mathcal {I}}(t),t>0$ has a unique point of minimum.

The proof of Lemma A.3 is purely analytical, thus we skip the details, referring for precise argumentation to the extended version of this contribution (Dȩbicki et al. 2021).

### **Lemma A.4**

Suppose that Σ = ΓΓ^⊤^ is positive definite. For any non-empty subset $\mathcal {I}\subset \{1,\ldots , d\}$ if $\boldsymbol {c}_{\mathcal {I}}$ and $\boldsymbol {a}_{\mathcal {I}}+\boldsymbol {c}_{\mathcal {I}} t$ for all *t* ≥ 0 have at least one positive component, then for any point $0<t\not =\hat {t}=\arg \min \limits _{t>0} r_{\mathcal {I}}(t)$ there exists some positive constant *ν* such that

$$ \begin{array}{@{}rcl@{}}\mathbb{P} \left\{ \boldsymbol{W}_{\mathcal{I}}(t)>(\boldsymbol {a}_{\mathcal{I}}+\boldsymbol{c}_{\mathcal{I}} t)\sqrt{u} \right \} =o\left( e^{-\nu u}\right)\mathbb{P} \left\{ \boldsymbol{W}_{\mathcal{I}}(\hat{t})>(\boldsymbol {a}_{\mathcal{I}}+\boldsymbol{c}_{\mathcal{I}}\hat{t})\sqrt{u} \right \} , \quad u\to \infty. \end{array} $$

### *Proof of Lemma A.4*

For notational simplicity we omit below the subscript $\mathcal {I}$. Since for any *t* > 0 we have *V* *a**r*(***W***(*t*)) = *t*Σ, then by Lemma 4.1

$$ \begin{array}{@{}rcl@{}} \mathbb{P} \left\{ \boldsymbol{W}(t)>(\boldsymbol {a}+\boldsymbol ct)\sqrt{u} \right \} \sim Cu^{-\alpha(t)/2}e^{-\frac{u}{2t}\tilde{\boldsymbol{\mathfrak{p}}}(t)^{\top}{\Sigma}^{-1}\tilde{\boldsymbol{\mathfrak{p}}}(t)}, \end{array} $$

where *C* is some positive constant, *α*(*t*) is an integer and $\tilde {\boldsymbol {\mathfrak {p}}}(t)$ is the unique solution of π_*t*Σ_(***a*** + ***c****t*), which can be reformulated also as

$$ \begin{array}{@{}rcl@{}} \mathbb{P} \left\{ \boldsymbol{W}(t)>(\boldsymbol {a}+\boldsymbol ct)\sqrt{u} \right \} \sim Cu^{-\alpha(t)/2}e^{-\frac{u}{2}r(t)}, \quad u\to \infty. \end{array} $$

If $t\not =\hat {t}$, then $r(t)-r(\hat {t})=\tau >0$ and

$$ \begin{array}{@{}rcl@{}} \frac{\mathbb{P} \left\{ \boldsymbol{W}(t)>(\boldsymbol {a}+\boldsymbol ct)\sqrt{u} \right \} }{\mathbb{P} \left\{ \boldsymbol{W}(\hat{t})>(\boldsymbol {a}+\boldsymbol{c}\hat{t})\sqrt{u} \right \} }\sim C^{*}u^{(\alpha(\hat{t})-\alpha(t))/2}e^{-\frac{\tau u}{2}}=o\left( e^{-\frac{\tau}{3}u}\right) \end{array} $$

as $u\to \infty $. □

### **Lemma A.5**

Let $\boldsymbol {a},\boldsymbol {c}\in \mathbb {R}^{d}$ be such that ***a*** + ***c****t* has at least one positive component for all *t* in a compact set $\mathcal {T}\subset (0, \infty )$. If Σ = ΓΓ^⊤^ is positive definite, then there exist constants *C* > 0, *γ* > 0 and $\mathfrak {t}\in \mathcal {T}$ such that for all *u* > 0

$$ \begin{array}{@{}rcl@{}}\mathbb{P} \left\{ \exists t\in\mathcal{T}:~\boldsymbol{W}(t)>(\boldsymbol {a}+\boldsymbol{c} t)\sqrt{u} \right \} \leq Cu^{\gamma}e^{-\frac{u}{2}r(\mathfrak{t})}. \end{array} $$

If we also have that for some non-overlapping index sets $\mathcal {I},\mathcal {J}\subset \{1,\ldots , d\}$ and some compact subset $\mathcal {T}\subset [0,\infty )^{2}$ both $((\boldsymbol {a}_{\mathcal {I}}+\boldsymbol {c}_{\mathcal {I}} t_{1})^{\top }, (\boldsymbol {a}_{\mathcal {J}}+\boldsymbol {c}_{\mathcal {J}} t_{2})^{\top })^{\top }$ have at least one positive component for all $(t_{1},t_{2})\in \mathcal {T}$, then for some $\mathfrak {t}=(\mathfrak {t}_{1},\mathfrak {t}_{2})\in \mathcal {T}$ as $u\to \infty $

$$ \begin{array}{@{}rcl@{}} && \mathbb{P} \left\{ \exists t\in\mathcal{T}:~\boldsymbol{W}_{\mathcal{I}}(t_1)>(\boldsymbol {a}_{\mathcal{I}}+\boldsymbol{c}_{\mathcal{I}} t_1)\sqrt{u},~\boldsymbol{W}_{\mathcal{J}}(t_{{2}})>(\boldsymbol {a}_{\mathcal{J}}+\boldsymbol{c}_{\mathcal{J}} t_2)\sqrt{u} \right \} \\ && \qquad\qquad= o(e^{\sqrt{u}}\mathbb{P} \left\{ \boldsymbol{W}_{\mathcal{I}}({\mathfrak{t}}_1)>(\boldsymbol {a}_{\mathcal{I}}+\boldsymbol{c}_{\mathcal{I}} \mathfrak{t}_1)\sqrt{u}, ~\boldsymbol{W}_{\mathcal{J}}({\mathfrak{t}_2})>(\boldsymbol {a}_{\mathcal{J}}+\boldsymbol{c}_{\mathcal{J}} {\mathfrak{t}_2})\sqrt{u} \right \} ). \end{array} $$

Moreover, the same estimate holds if $\mathcal {I}$ and $\mathcal {J}$ are overlapping and for all $(\mathfrak {t}_{1},\mathfrak {t_{2}})\in \mathcal {T}$ we have $\mathfrak {t}_{1}\not =\mathfrak {t}_{2}$.

### *Proof of Lemma A.5*

Denote by *D*(*t*) the covariance matrix of ***W***(*t*), which by assumption on Γ is positive definite. Let $\tilde {\mathfrak {a}}(t) = \arg \min \limits _{\boldsymbol {x}\geq \boldsymbol {a}+\boldsymbol {c} t} \boldsymbol {x}^{\top } D^{-1}(t) \boldsymbol {x}$ be the solution of π_*D*_(***a*** + ***c****t*), *t* > 0 and let further

$$ \begin{array}{@{}rcl@{}}\mathfrak{w}(t) =D^{-1}(t)\tilde{\mathfrak{a}}(t) \end{array} $$

be the solution of the dual optimization problem. In view of Eq. 10$\mathfrak {w}_{I}(t)$ has positive components for *I* the unique index set related to π_*D*(*t*)_(***a*** + ***c****t*) and moreover by Eq. 9

$$f(t)=\mathfrak{w}^{\top}(t) (\boldsymbol {a}+ \boldsymbol{c} t) = \tilde{\mathfrak{a}}^{\top}(t) D^{-1}(t)\tilde{\mathfrak{a}}(t)>0 $$

implying

$$ \begin{array}{@{}rcl@{}}\mathbb{P} \left\{ \exists t\in\mathcal{T}:\boldsymbol{W}(t)\geq(\boldsymbol {a}+\boldsymbol{c} t) \sqrt{u} \right \} &\leq& \mathbb{P} \left\{ \exists t\in\mathcal{T}:\frac{\mathfrak{w}^{\top} {(t)} \boldsymbol{W}(t)}{\mathfrak{w}^{\top} {(t)}(\boldsymbol {a}+\boldsymbol{c} t)}\geq \sqrt{u} \right \} . \end{array} $$

We have further that

$$ \begin{array}{@{}rcl@{}}\sigma^{2}=\sup_{t\in\mathcal{T}}\mathbb{E}\left\{ \left( \frac{\mathfrak{w}^{\top}(t)\boldsymbol{W}(t)}{\mathfrak{w}^{\top}(t) (\boldsymbol {a}+\boldsymbol{c} t)}\right)^2\right\} =\sup_{t\in\mathcal{T}}\frac{1}{\tilde{\mathfrak{a}}^{\top}(t) D^{-1}(t)\tilde{\mathfrak{a}}(t)} =\frac{1}{\tilde{\mathfrak{a}}^{\top}(\mathfrak{t})D^{-1}(\mathfrak{t})\tilde{\mathfrak{a}}(\mathfrak{t})}>0 \end{array} $$

for some $\mathfrak {t}\in \mathcal {T}$, since $\mathcal {T}$ is compact. Since $f(t)>0, t\in \mathcal {T}$ is continuous, we may apply Piterbarg inequality (as in the proof of Eq. 20) and obtain

$$ \begin{array}{@{}rcl@{}}\mathbb{P} \left\{ \exists t\in\mathcal{T}:\boldsymbol{W}(t)\geq(\boldsymbol {a}+\boldsymbol ct)\sqrt{u} \right \} \leq Cu^{\gamma} e^{-u/2\sigma^2} \end{array} $$

for some positive constants *γ* and *C*, which depend only on ***W***(*t*) and *d*. Since, by the definition we have $r(\mathfrak {t})=1/\sigma ^{2}$, the proof of the first inequality is complete.

The next assertion may be obtained with the same arguments but for vector-valued random process

$$ \begin{array}{@{}rcl@{}}\mathcal{W}(s,t)=(\boldsymbol{W}_{\mathcal{I}}^{\top}(s),\boldsymbol{W}_{\mathcal{J}}^{\top}(t))^{\top}. \end{array} $$

By the definition of $\mathcal {T}$, for any $(s,t)\in \mathcal {T}$ we have $\lvert Var(\mathcal {W}(s,t))\rvert >0$, thus we can apply Piterbarg inequality and in consequence, using Lemma 4.1, the claim follows. □

### **Lemma A.6**

Suppose that Σ = ΓΓ^⊤^ is positive definite. For any subset $\mathcal {I}\subset \{1,\ldots , d\}$ if $\boldsymbol {c}_{\mathcal {I}} \in \mathbb {R}^{\left \lvert \mathcal {I} \right \rvert }$ has at least one positive component and $\boldsymbol {a}_{\mathcal {I}}+\boldsymbol {c}_{\mathcal {I}} t \in \mathbb {R}^{\left \lvert {\mathcal {I}} \right \rvert }$ has at least one positive component for all non-negative *t*, then for some positive constants *ν*, $\hat {t}=\arg \min \limits _{t>0} r_{\mathcal {I}}(t)$ and all *T* large

$$ \mathbb{P} \left\{ \exists t>T:~\boldsymbol{W}_{\mathcal{I}}(t)>(\boldsymbol {a}_{\mathcal{I}}+\boldsymbol{c}_{\mathcal{I}} t)\sqrt{u} \right \} =o(e^{-\nu {u}}) \mathbb{P} \left\{ \boldsymbol{W}_{\mathcal{I}}(\hat{t})>(\boldsymbol {a}_{\mathcal{I}}+\boldsymbol{c}_{\mathcal{I}} \hat{t}){\sqrt{u}} \right \} ,\qquad u\to\infty. $$

### *Proof of Lemma A.6*

For notational simplicity we omit below the subscript $\mathcal {I}$. For some given $T>\hat {t} $ we have using Lemmas A.5, A.3

$$ \begin{array}{@{}rcl@{}} \mathbb{P} \left\{ \exists t>T:~\boldsymbol{W}(t)>(\boldsymbol {a}+\boldsymbol ct)\sqrt{u} \right \} &\leq& {\sum}_{i=0}^{\infty}\mathbb{P} \left\{ \exists t\in [T+i,T+i+1]:~\boldsymbol{W}(t)>(\boldsymbol {a}+\boldsymbol ct)\sqrt{u} \right \} \\ &\leq&{\sum}_{i=0}^{\infty}Cu^{\gamma}e^{-\frac{r(\mathfrak{t}_i)}{2}u}\\ &\leq & Cu^{\gamma}e^{-\frac{r(T)}{2}u}{\sum}_{i=0}^{\infty}e^{-isu} \\ &\leq& Cu^{\gamma}e^{-\frac{r(T)}{2}u}\left( 1+{\int}_{0}^{\infty} e^{-sux}\mathrm{d} x\right), \end{array} $$

where *s* > 0 and *t*_*i*_ ∈ [*T* + *i*, *T* + *i* + 1]. The last integral is finite and decreasing for sufficiently large *u*. Hence the claim follows with the same arguments as in the proof of Lemma A.4. □

### *Proof of Lemma 4.4*

Using Lemma A.6 we know that there exist points $t_{\mathcal {I}},~t_{\mathcal {J}}$ such that

$$\mathbb{P} \left\{ \exists t\geq T_{\mathcal{I}}:~A^{*}_{\mathcal{I}}(t) \right \} = o(\mathbb{P} \left\{ A^{*}_{\mathcal{I}}(\hat{t}_{\mathcal{I}}) \right \} ),\qquad \mathbb{P} \left\{ \exists t\geq T_{\mathcal{J}}:~A^{*}_{\mathcal{J}}(t) \right \} =o(\mathbb{P} \left\{ A^{*}_{\mathcal{J}}(\hat{t}_{\mathcal{J}}) \right \} ),\qquad u\to\infty. $$

Next, for some positive $\varepsilon <|\hat {t}_{\mathcal {I}}-\hat {t}_{\mathcal {J}}|/3$ we have

$$ \begin{array}{@{}rcl@{}} \mathbb{P} \left\{ \exists s,t>0:~A^{*}_{\mathcal{I}}(t) {\cap} A^{*}_{\mathcal{J}}(s) \right \} &\leq&\mathbb{P} \left\{ \exists (s,t)\in[\hat{t}_{\mathcal{I}}-\varepsilon,\hat{t}_{\mathcal{I}}+\varepsilon]\times[\hat{t}_{\mathcal{J}}-\varepsilon,\hat{t}_{\mathcal{J}}+\varepsilon]:~A^{*}_{\mathcal{I}}(t)\cap A^{*}_{\mathcal{J}}(s) \right \} \\ &+&\mathbb{P} \left\{ \exists t\in[0,\hat{t}_{\mathcal{I}}-\varepsilon]:~A^{*}_{\mathcal{I}}(t) \right \} +\mathbb{P} \left\{ \exists t\in[\hat{t}_{\mathcal{I}}+\varepsilon,T_{\mathcal{I}}]:~A^{*}_{\mathcal{I}}(t) \right \} \\ &+&\mathbb{P} \left\{ \exists t\in[0,\hat{t}_{\mathcal{J}}-\varepsilon]:~A^{*}_{\mathcal{J}}(t) \right \} +\mathbb{P} \left\{ \exists t\in[\hat{t}_{\mathcal{J}}+\varepsilon,T_{\mathcal{J}}]:~A^{*}_{\mathcal{J}}(t) \right \} \\ &+&\mathbb{P} \left\{ \exists t\geq T_{\mathcal{I}}:~A^{*}_{\mathcal{I}}(t) \right \} +\mathbb{P} \left\{ \exists t\geq T_{\mathcal{J}}:~A^{*}_{\mathcal{J}}(t) \right \} . \end{array} $$

Using Lemmas A.5, A.6 and

$$\mathbb{P} \left\{ A^{*}_{\mathcal{I}}(t) \right \} \sim Cu^{-\alpha}e^{-r(t)u/2}, \quad \mathbb{P} \left\{ A^{*}_{\mathcal{I}}(t) \right \} =o(ue^{-r(t)u/2}), \quad u\to \infty$$

we obtain

$$ \begin{array}{@{}rcl@{}} \mathbb{P} \left\{ \exists s,t>0:~A^{*}_{\mathcal{I}}(t){\cap} A^{*}_{\mathcal{J}}(s) \right \} &{=}& o(e^{\sqrt{u}} \mathbb{P} \left\{ A^{*}_{\mathcal{I}}(s_1)\cap A^{*}_{\mathcal{J}}(s_2) \right \} )\\ &+&o(u^{\tau_3}\mathbb{P} \left\{ A^{*}_{\mathcal{I}}(t_3) \right \} )+o(u^{\tau_4}\mathbb{P} \left\{ A^{*}_{\mathcal{I}}(t_4) \right \} )\\ &+&o(u^{\tau_5}\mathbb{P} \left\{ A^{*}_{\mathcal{J}}(t_5) \right \} )+o(u^{\tau_6}\mathbb{P} \left\{ A^{*}_{\mathcal{J}}(t_6) \right \} )\\ &+&o(\mathbb{P} \left\{ A^{*}_{\mathcal{I}}(\hat{t}_{\mathcal{I}}) \right \} )+o(\mathbb{P} \left\{ A^{*}_{\mathcal{J}}(\hat{t}_{\mathcal{J}}) \right \} ) \end{array} $$

for some positive constants *t*_*i*_, 3 ≤ *i* ≤ 6, where

$$t_3\in[0,\hat{t}_{\mathcal{I}}-\varepsilon],\quad t_4\in[\hat{t}_{\mathcal{I}}+\varepsilon,T_{\mathcal{I}}], \quad t_5\in[0,\hat{t}_{\mathcal{J}}-\varepsilon],\quad t_6\in[\hat{t}_{\mathcal{J}}+\varepsilon,T_{\mathcal{J}}]\quad s_1\in[\hat{t}_{\mathcal{I}}-\varepsilon,\hat{t}_{\mathcal{I}}+\varepsilon]\quad s_2\in[\hat{t}_{\mathcal{J}}-\varepsilon,\hat{t}_{\mathcal{J}}+\varepsilon]. $$

Note that for *i* = 3, 4, $t_{i}\not =\hat {t}_{\mathcal {I}}$. Hence by Lemma A.4

$$ \begin{array}{@{}rcl@{}}u^{\tau_i}\mathbb{P} \left\{ A^{*}_{\mathcal{I}}(t_i) \right \} =o(\mathbb{P} \left\{ A^{*}_{\mathcal{I}}(\hat{t}_{\mathcal{I}}) \right \} ). \end{array} $$

The same works also for *j* = 5, 6

$$ \begin{array}{@{}rcl@{}} u^{\tau_j}\mathbb{P} \left\{ A^{*}_{\mathcal{J}}(t_j) \right \} =o(\mathbb{P} \left\{ A^{*}_{\mathcal{J}}(\hat{t}_{\mathcal{J}}) \right \} ). \end{array} $$

Thus we can focus only on the first probability. By the definition of $A^{*}_{\mathcal {I}}$ and $A^{*}_{\mathcal {J}}$ in Eq. 21

$$ \begin{array}{@{}rcl@{}}\mathbb{P} \left\{ A^{*}_{\mathcal{I}}(s_1)\cap A^{*}_{\mathcal{J}}(s_2) \right \} =\mathbb{P} \left\{ \mathcal{W}(s_1,s_2) > \boldsymbol b \sqrt{u} \right \} , \end{array} $$

where $\boldsymbol b= ((\boldsymbol {a}_{\mathcal {I}}+\boldsymbol {c}_{\mathcal {I}} s_{1})^{\top },(\boldsymbol {a}_{\mathcal {J}}+\boldsymbol {c}_{\mathcal {J}} s_{2})^{\top })$ and $\mathcal {W}(s,t)=(\boldsymbol {W}_{\mathcal {I}}(s)^{\top },\boldsymbol {W}_{\mathcal {J}}(t)^{\top })^{\top }. $ Define $\widehat {i}=\mathcal {I}\cup \mathcal {J}\setminus \{i\}$. Applying Remark A.2, there exists an index *i* and a constant *η* > 0 such that

$$\mathbb{P} \left\{ A^{*}_{\mathcal{I}}(s_1)\cap A^{*}_{\mathcal{J}}(s_2) \right \} =o\left( e^{-\eta u}\right)\mathbb{P} \left\{ (\mathcal{W}(s_1,s_2))_{\widehat{i}} > \boldsymbol b_{\widehat{i}} \sqrt{u} \right \} . $$

If $i\in \mathcal {I}$, then

$$\mathbb{P} \left\{ (\mathcal{W}(s_1,s_2))_{\widehat{i}}>\boldsymbol b_{\widehat{i}}\sqrt{u} \right \} \leq\mathbb{P} \left\{ \boldsymbol{W}_{\mathcal{J}}(s_2)>(\boldsymbol {a}_{\mathcal{J}}+\boldsymbol{c}_{\mathcal{J}} s_2)u \right \} , $$

or

$$\mathbb{P} \left\{ (\mathcal{W}(s_1,s_2))_{\widehat{i}} > \boldsymbol b_{\widehat{i}} \sqrt{u} \right \} \leq\mathbb{P} \left\{ \boldsymbol{W}_{\mathcal{I}}(s_1)>(\boldsymbol {a}_{\mathcal{I}}+\boldsymbol{c}_{\mathcal{I}} s_1)u \right \} . $$

In both casess

$$ \begin{array}{@{}rcl@{}}e^{\sqrt{u}}\mathbb{P} \left\{ {A^{*}_{\mathcal{I}}(s_1)\cap A^{*}_{\mathcal{J}}(s_2)} \right \}&=&o\left( \mathbb{P} \left\{ {\boldsymbol{W}_{\mathcal{I}}(s_1)>(\boldsymbol {a}_{\mathcal{I}}+\boldsymbol{c}_{\mathcal{I}} s_1)u} \right \}+\mathbb{P} \left\{ {\boldsymbol{W}_{\mathcal{J}}(s_1)>(\boldsymbol {a}_{\mathcal{J}}+\boldsymbol{c}_{\mathcal{J}} s_1)u} \right \}\right)\\ &=&o\left( \mathbb{P} \left\{ \boldsymbol{W}_{\mathcal{I}}(\hat{t}_{\mathcal{I}})>(\boldsymbol {a}_{\mathcal{I}}+\boldsymbol{c}_{\mathcal{I}} \hat{t}_{\mathcal{I}})u \right \} +\mathbb{P} \left\{ \boldsymbol{W}_{\mathcal{J}}(\hat{t}_{\mathcal{I}})>(\boldsymbol {a}_{\mathcal{J}}+\boldsymbol{c}_{\mathcal{J}} \hat{t}_{\mathcal{J}})u \right \} \right) \end{array} $$

establishing the proof. □

### *Proof of Lemma 4.5*

Using Lemma A.6 we have

$$ \begin{array}{@{}rcl@{}} \mathbb{P} \left\{ \exists s,t>0:~A^{*}_{\mathcal{I}}(s){\cap} A^{*}_{\mathcal{J}}(t) \right \} &\leq& \mathbb{P} \left\{ \exists (s,t)\in\mathbb{T}_1:~A^{*}_{\mathcal{I}}(s){\cap} A^{*}_{\mathcal{J}}(t) \right \} +\mathbb{P} \left\{ \exists (s,t)\in\mathbb{T}_2:~A^{*}_{\mathcal{I}}(s){\cap} A^{*}_{\mathcal{J}}(t) \right \} \\ &+&o(\mathbb{P} \left\{ A^{*}_{\mathcal{I}}(\hat{t}_{\mathcal{I}}) \right \} )+o(\mathbb{P} \left\{ A^{*}_{\mathcal{J}}(\hat{t}_{\mathcal{J}}) \right \} ), \end{array} $$

where

$$ \begin{array}{@{}rcl@{}} \mathbb{T}_1&=&\{(s,t)\in[0,T_{\mathcal{I}}]\times[0,T_{\mathcal{J}}]:|s-\hat{t}_{\mathcal{I}}|\geq|t-\hat{t}_{\mathcal{I}}|\},\\ \mathbb{T}_2&=&\{(s,t)\in[0,T_{\mathcal{I}}]\times[0,T_{\mathcal{J}}]:|s-\hat{t}_{\mathcal{I}}|\leq|t-\hat{t}_{\mathcal{I}}|\} \end{array} $$

and $T_{\mathcal {I}}$ and $T_{\mathcal {J}}$ are the constants from Eq. 29. According to Lemma A.5 for some $(s_{i},t_{i})\in \mathbb {T}_{i}$

$$\mathbb{P} \left\{ \exists (s,t)\in\mathbb{T}_i:A^{*}_{\mathcal{I}}(s)\cap A^{*}_{\mathcal{J}}(t) \right \} =o \Bigl(e^{\sqrt{u}} \Bigr) \mathbb{P} \left\{ A^{*}_{\mathcal{I}}(s_i)\cap A^{*}_{\mathcal{J}\setminus\mathcal{I}}(t_i) \right \} . $$

If $s_{1}\not =\hat {t}_{\mathcal {I}}$, then according to Lemma A.4

$$e^{\sqrt{u}}\mathbb{P} \left\{ A^{*}_{\mathcal{I}}(s_1)\cap A^{*}_{\mathcal{J}\setminus\mathcal{I}}(t_1) \right \} \leq e^{\sqrt{u}}\mathbb{P} \left\{ A^{*}_{\mathcal{I}}(s_1) \right \} =o(\mathbb{P} \left\{ A^{*}_{\mathcal{I}}(\hat{t}_{\mathcal{I}}) \right \} ). $$

Otherwise, using the definition of $\mathbb {T}_{1}$, $|t_{1}-\hat {t}_{\mathcal {I}}|\leq |s_{1}-\hat {t}_{\mathcal {I}}|=0$, so $t_{1}=\hat {t}_{\mathcal {I}}$ and thus

$$\mathbb{P} \left\{ A^{*}_{\mathcal{I}}(s_1)\cap A^{*}_{\mathcal{J}\setminus\mathcal{I}}(t_1) \right \} =\mathbb{P} \left\{ A^{*}_{\mathcal{I}\cup\mathcal{J}}(\hat{t}_{\mathcal{I}}) \right \} . $$

This probability can be bounded using Remark A.2, namely we have

$$\mathbb{P} \left\{ A^{*}_{\mathcal{I}\cup\mathcal{J}}(\hat{t}_{\mathcal{I}}) \right \} =o\Bigl(e^{-\nu {u}} \Bigr)\mathbb{P} \left\{ A^{*}_{\mathcal{I}\cup\mathcal{J}\setminus\{i\}}(\hat{t}_{\mathcal{I}}) \right \} $$

for some $i\in \mathcal {I}\cup \mathcal {J}$ and *η* > 0. As $|\mathcal {I}|=|\mathcal {J}|=k$, and $\mathcal {I}\not =\mathcal {J}$, then $|\mathcal {I}\cup \mathcal {J}|\ge k+1$ and thus $|\mathcal {I}\cup \mathcal {J}\setminus \{i\}|\ge k$. Consequently, we have

$$ e^{\sqrt{u}}\mathbb{P} \left\{ A^{*}_{\mathcal{I}\cup\mathcal{J}}(\hat{t}_{\mathcal{I}}) \right \} =o\left( \mathbb{P} \left\{ A^{*}_{\mathcal{I}\cup\mathcal{J}\setminus\{i\}}(\hat{t}_{\mathcal{I}}) \right \} \right)=o\left( \underset{|\mathcal{K}|=k}{\sum\limits_{\mathcal{K}\subset\{1\ldots d\}}}\mathbb{P} \left\{ A^{*}_{\mathcal{K}}(\hat{t}_{\mathcal{K}}) \right \} \right). $$

With similar arguments we obtain further

$$ \mathbb{P} \left\{ \exists (s,t)\in\mathbb{T}_2:~A^{*}_{\mathcal{I}}(s){\cap} A^{*}_{\mathcal{J}}(t) \right \} =o\left( \underset{|\mathcal{K}|=k}{\sum\limits_{\mathcal{K}\subset\{1{\ldots} d\}}} \mathbb{P} \left\{ A^{*}_{\mathcal{K}}(\hat{t}_{\mathcal{K}}) \right \} \right). $$

Hence the claim follows. □

Recall that $\tilde { \boldsymbol {a}}$ stands for the unique solution of the quadratic programming problem π_Σ_(***a***).

### *Proof of Lemma 4.6*

By the self-similarity of Brownian motion for all *u* > 0

$$m(u,{\Lambda}):=\mathbb{P} \left\{ \exists_{t\in[0,\delta(u,{\Lambda})]}:\boldsymbol{W}(t)-t\boldsymbol{c}> u\boldsymbol {a} \right \} =\mathbb{P} \left\{ \exists_{t\in[0,1]}:\boldsymbol{W}(t)-\delta^{1/2}(u,{\Lambda})t\boldsymbol{c}> \delta^{-1/2}(u,{\Lambda})u\boldsymbol {a} \right \} . $$

Hence, applying Theorem 1.1 we obtain

$$ m(u,{\Lambda})\le\frac{\mathbb{P} \left\{ \boldsymbol{W}(1)\ge \delta^{-1/2}(u,{\Lambda})u\boldsymbol {a} +\delta^{1/2}(u,{\Lambda})\boldsymbol{c} \right \} }{\mathbb{P} \left\{ \boldsymbol{W}(1)>\max(\boldsymbol{c},\boldsymbol 0) \right \} }, $$

which after some standard algebraic manipulations, straightforwardly implies inequality (23).

Equation 24 and limit (25) follow by the same idea as the proof of “Pickands’ lemma” in e.g. Dȩbicki et al. (2018); see Lemmas 4.2 and 4.3 therein. We skip long but standard proof, referring for details to the extended version of this contribution (Dȩbicki et al. 2021). □

## References

[CR1] Avram F, Palmowski Z, Pistorius M (2008). A two-dimensional ruin problem on the positive quadrant. Insurance Math. Econom..

[CR2] Avram F, Palmowski Z, Pistorius MR (2008). Exit problem of a two-dimensional risk process from the quadrant: exact and asymptotic results. Ann. Appl. Probab..

[CR3] Bai L, Dȩbicki K, Liu P (2018). Extremes of vector-valued Gaussian processes with trend. J. Math. Anal. Appl..

[CR4] Borovkov, K., Palmowski, Z.: The Exact Asymptotics for Hitting Probability of a Remote Orthant by a Multivariate Lévy Process: the Cramé Case. In: 2017 MATRIX Annals vol. 2 of MATRIX Book Ser, pp 303–309. Springer, Cham (2019)

[CR5] Dȩbicki K, Hashorva E, Ji L, Rolski T (2018). Extremal behavior of hitting a cone by correlated Brownian motion with drift. Stoch. Proc. Appl..

[CR6] Dȩbicki, K., Hashorva, E., Kriukov, N.: Pandemic-type failures in multivariate Brownian risk models. arXiv:2008.07480 (2021) 10.1007/s10687-021-00424-4 PMC8816775 35221783

[CR7] Dȩbicki K, Hashorva E, Liu P (2017). Extremes of γ-reflected gaussian processes with stationary increments. ESAIM Probab. Stat..

[CR8] Dȩbicki K, Hashorva E, Michna Z (2020). Simultaneous ruin probability for two-dimensional Brownian risk model. J. Appl. Probab..

[CR9] Delsing GA, Mandjes MRH, Spreij PJC, Winands EMM (2020). Asymptotics and approximations of ruin probabilities for multivariate risk processes in a Markovian environment. Methodol. Comput. Appl. Probab..

[CR10] Dombry C, Rabehasaina L (2017). High order expansions for renewal functions and applications to ruin theory. Ann. Appl. Probab..

[CR11] Foss S, Korshunov D, Palmowski Z, Rolski T (2017). Two-dimensional ruin probability for subexponential claim size. Probab. Math. Statist..

[CR12] Hashorva E (2019). Approximation of some multivariate risk measures for Gaussian risks. J. Multivariate Anal..

[CR13] Hu Z, Jiang B (2013). On joint ruin probabilities of a two-dimensional risk model with constant interest rate. J. Appl. Probab..

[CR14] Ji L (2020). On the cumulative Parisian ruin of multi-dimensional Brownian motion risk models. Scand. Actuar. J..

[CR15] Ji L, Robert S (2018). Ruin problem of a two-dimensional fractional Brownian motion risk process. Stoch. Models.

[CR16] Korshunov D, Wang L (2020). Tail asymptotics for Shepp-statistics of Brownian motion in $\mathbb {R}^{d}$ℝd. Extremes.

[CR17] Korshunov DA, Piterbarg VI, Hashorva E (2015). On the asymptotic Laplace method and its application to random chaos. Mat. Zametki.

[CR18] Lieshout P, Mandjes M (2007). Tandem Brownian queues. Math. Methods Oper. Res..

[CR19] Pan Y, Borovkov KA (2019). The exact asymptotics of the large deviation probabilities in the multivariate boundary crossing problem. Adv. Appl. Probab..

[CR20] Piterbarg, V.I.: Asymptotic Methods in the Theory of Gaussian Processes and Fields, Vol. 148 of Translations of Mathematical Monographs. Providence, RI, American Mathematical Society. Translated from the Russian by V.V. Piterbarg, revised by the author (1996)

[CR21] Samorodnitsky G, Sun J (2016). Multivariate subexponential distributions and their applications. Extremes.

